# Circulating tumor DNA monitoring and blood tumor mutational burden in patients with metastatic solid tumors treated with atezolizumab

**DOI:** 10.1002/1878-0261.70054

**Published:** 2025-05-28

**Authors:** Charles Swanton, Russell W. Madison, Candice Francheska B. Tambaoan, Funda Meric‐Bernstam, Christopher J. Sweeney, Razelle Kurzrock, Howard A. Burris, David R. Spigel, Hanna Tukachinsky, Jason Hughes, Julia Malato, Bongin Yoo, Tania Szado, Cheryl Schwab, Lincoln W. Pasquina, Amaya Gasco, Katja Schulze, Claire F. Friedman

**Affiliations:** ^1^ Cancer Evolution and Genome Instability Laboratory The Francis Crick Institute London UK; ^2^ Department of Oncology University College London Hospitals London UK; ^3^ Foundation Medicine, Inc. Boston MA USA; ^4^ Department of Investigational Cancer Therapeutics The University of Texas MD Anderson Cancer Center Houston TX USA; ^5^ University of Adelaide Adelaide Australia; ^6^ Department of Medicine Medical College of Wisconsin Milwaukee WI USA; ^7^ Sarah Cannon Research Institute Nashville TN USA; ^8^ Genentech, Inc. South San Francisco CA USA; ^9^ Memorial Sloan Kettering Cancer Center New York NY USA; ^10^ Department of Medicine Weill Cornell Medicine New York NY USA

**Keywords:** ctDNA tumor fraction, immune checkpoint inhibitors, tumor mutational burden

## Abstract

Immune checkpoint inhibitors are important for treatment across tumor types but are not universally effective in controlling disease. Early understanding of tumor response, or lack thereof, can inform treatment decisions. This study evaluates changes in circulating tumor DNA (ctDNA) and blood tumor mutational burden (bTMB) for associations with response to programmed cell death 1 ligand 1 (PD‐L1) blockade. We sequenced cell‐free DNA collected at the start of therapy, on treatment, and at the end of therapy for 153 patients treated with atezolizumab as part of the pan‐tumor MyPathway study (NCT02091141). ctDNA tumor fraction (TF) and bTMB were assessed for correlation with progression‐free survival (PFS) and overall survival (OS). We found that molecular response (MR, ≥50% decrease in TF at cycle 3 day 1) was associated with improved PFS (9.7 vs 1.5 months from C3D1; HR = 0.27) and OS (21.1 vs 14.3 months from C3D1; HR = 0.44). These findings were consistent when limited to patients with stable disease (SD; PFS HR = 0.55; OS HR = 0.39). bTMB was correlated with tissue‐based TMB (tTMB) when TF was high (≥1%), but not with OS in this cohort. In total, 61% of baseline samples had predicted clonal hematopoiesis (CH) variants. No correlation between maximum variant allele frequency (maxVAF) of predicted CH and TF was seen. In summary, MR is associated with outcomes for patients treated with atezolizumab and could stratify patients with SD. While CH was common, maxVAF for CH variants was not associated with ctDNA TF. Quantification of ctDNA enables therapy response monitoring and is critical for interpretation of bTMB as a proxy for tTMB.

## Graphical abstract

In patients treated with atezolizumab as a part of the MyPathway (NCT02091141) trial, pre‐treatment ctDNA tumor fraction at high levels was associated with poor outcomes (radiographic response, progression‐free survival, and overall survival) but better sensitivity for blood tumor mutational burden (bTMB). Persistent negative ctDNA tumor fraction or substantial decreases in ctDNA tumor fraction (molecular response) were associated with improved outcomes.

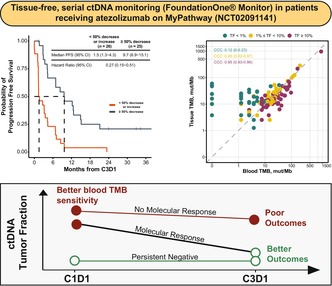

**Abbreviations**
bTMB: blood tumor mutational burden
C1D1: cycle one day one
C3D1: cycle three day one
cBOR: confirmed best overall response
CCC: concordance correlation coefficient
cfDNA: cell‐free DNA
CH: clonal hematopoiesis
CR: complete response
CRC: colorectal carcinoma
ctDNA: circulating tumor DNA
CUP: cancer of unknown primary
DCR: disease control rate
EOT: end of treatment
F1LCDx: FoundationOneLiquid CDx
HR: Hazard ratio
ICI: immune checkpoint inhibitor
IHC: immunohistochemistry
IQR: interquartile range
maxVAF: maximum variant allele frequency
MR: molecular response
MSI: microsatellite instability
MSI‐H: microsatellite instability high
mut/Mb: mutations per megabase
NSCLC: non‐small cell lung cancer
ORR: overall response rate
OS: overall survival
PD: progressive disease
PD‐L1: programmed death‐ligand 1
PFS: progression‐free survival
PR: partial response
RECIST: Response Evaluation Criteria in Solid Tumors
SD: stable disease
SLD: sum of target lesions longest diameter
TF: tumor fraction
tTMB: tissue tumor mutational burden
VOP: variant origin prediction

## 1 Introduction

Immune checkpoint inhibitor (ICI) therapy has been a groundbreaking development in the treatment of cancer across solid tumors, but response is not universal [1, 2]. Rapid, reliable assessments of response could aid decisions among the growing variety of therapies, combinations, doses, and regimens available across tumor types. Identification of predictive biomarkers such as programmed death‐ligand 1 (PD‐L1) expression, microsatellite instability (MSI), and tumor mutational burden (TMB) helps guide ICI use, but need remains in further refining treatment strategies before and during treatment [3, 4, 5].

Circulating tumor DNA (ctDNA) is prognostic in patients with solid tumors and has emerged as a powerful tool for assessing response to therapy [6, 7, 8, 9, 10, 11]. In patients treated with ICI, ctDNA monitoring can provide additional value to the interpretation of radiographic imaging, which may be confounded by pseudo‐progression or otherwise inconclusive [12, 13]. Additionally, tissue is not always readily available for assessing biomarkers predictive of ICI response. Studies have demonstrated that blood tumor mutational burden (bTMB) found in baseline ctDNA is correlated with TMB assessed from tissue biopsy (tTMB), suggesting bTMB can serve as a surrogate when tissue is unavailable for TMB assessment [14, 15]. The B‐FIRST trial and retrospective analysis from the OAK and POPLAR trials showed benefit for non‐small cell lung cancer patients treated with atezolizumab whose baseline bTMB was elevated [16, 17]. However, detection of bTMB is dependent on the sample containing sufficient ctDNA tumor fraction (TF) [15]. The interaction between the predictive value of bTMB vs ctDNA TF as both a prognostic factor and indicator of sensitivity has not been adequately described in the context of ICI treatment.

MyPathway, a multicenter, non‐randomized, open‐label phase IIa basket study, evaluated multiple targeted therapies in patients with advanced solid tumors with potentially actionable alterations [18]. In one cohort, atezolizumab was assessed in patients with solid tumors without an FDA approval for treatment with the study drug. Significant improvement in both PFS and OS was seen for those with tTMB ≥16 mutations per megabase (mut/Mb) compared to those with tTMB ≥10 mut/Mb but <16 mut/Mb [19].

Here we report a retrospective analysis of plasma samples collected from patients treated with atezolizumab as part of the MyPathway trial. We investigated both ctDNA dynamics and bTMB as markers of response in a pan‐tumor cohort as well as the relationship between these two blood‐based biomarkers.

## 2 Materials and methods

### 2.1 Study design and patient selection

The trial design of MyPathway (NCT02091141) has been previously described [19]. Briefly, adults whose tumors had tTMB ≥10 mut/Mb as determined locally by any CLIA‐certified assay were administered atezolizumab 1200 mg intravenously every 3 weeks until disease progression or unacceptable toxicity. Tumor size and response were assessed by the investigator per Response Evaluation Criteria in Solid Tumors (RECIST) version 1.1 at baseline and every two treatment cycles (every 6 weeks) for the first 24 weeks, and then every four treatment cycles (every 12 weeks) thereafter. PD‐L1 IHC testing was conducted using the clone 22C3 pharmDx kit and was reported as tumor proportion score (TPS). Patients not reported here had no local PD‐L1 test result and no tissue or insufficient tissue for central testing. Tumor burden was quantified using the sum of (target) lesions longest diameter (SLD). Patients were treated between July 2017 and May 2023. Plasma was collected at the start of treatment cycles 1 and 3 and at progression or end of treatment (EOT). To minimize bias, ctDNA measurements were conducted with blinding to clinical data (*e.g.*, sex and age).

MyPathway was conducted in accordance with the International Conference on Harmonization guideline for Good Clinical Practice and the Declaration of Helsinki. The protocol and study were approved by the institutional review board/ethics committee at each trial center. All patients provided written informed consent to participate in the study.

### 2.2 Cell‐free DNA sequencing and analysis

Samples were analyzed using FoundationOne® Monitor, a tissue‐agnostic ctDNA monitoring assay using hybrid capture next generation sequencing, leveraging the same assay platform as FoundationOne®Liquid CDx (F1LCDx), with methods described previously [20]. Median sequencing depth of non‐redundant reads for all analyzed liquid biopsy samples was 2056X (IQR: 1712‐2288X). The assay detects and quantifies the fraction of ctDNA of the total cell‐free DNA (cfDNA) present in a liquid biopsy sample as ctDNA TF, calculated as previously described [21, 22]. In brief, ctDNA TF in a sample is quantified by integrating multiple distinct signals, including aneuploidy, the presence of short variants, and cfDNA fragment metrics from the sample. Fragment information is used to limit the contribution of clonal hematopoiesis (CH) to the aneuploidy‐based estimate and to help identify tumor‐somatic short variants. The lowest ctDNA TF detected and quantified in this study was 0.2%. ctDNA TF change was not quantified when ctDNA TF was detected below 2% at both on C1D1 and C3D1.

Blood tumor mutational burden (bTMB) was calculated from baseline F1LCDx results by counting the number of single nucleotide variants detected at an allele frequency of ≥0.5%, after the removal of known and likely oncogenic driver events and germline single nucleotide polymorphisms, and dividing by the size of the baited coding regions (0.79 Mb) [16].

### 2.3 Tissue sequencing and analysis

Tissue samples were sequenced as available on FoundationOne® CDx to a median sequencing depth of 871X (IQR: 805‐969X) [23]. TMB from paired tissue specimens (tTMB) was calculated using FoundationOne® CDx as previously described [24]. All analyses of tissue and blood samples were performed independently, and bTMB and tTMB were compared for blood and tissue sample pairs from the same patient.

### 2.4 Variant origin predictions

We leveraged a variant origin prediction (VOP) machine learning model that, for all short variants (substitutions, small insertions, and deletions) detected in the F1LCDx assay, generates a 3‐way probability for whether the variant originated as: (1) germline, (2) CH, or (3) tumor somatic. This model was trained using a collection of 1977 F1LCDx results generated from plasma for which matched buffy coat DNA was also sequenced to equal depth. The model uses a variety of features, including fragmentomics (size and genomic positioning of cfDNA fragments supporting each short variant) and variant allele‐frequency‐related features in the context of somatic/germline expectations given a copy number model for the sample, but not including any patient demographics, which were combined and used to train a machine learning classification model.

### 2.5 Statistical analysis

Tumor response was assessed according to the guidelines of the RECIST 1.1 [25]. Overall response rate (ORR) was defined as the proportion of patients with the best overall response of complete or partial response; disease control rate (DCR) was defined the same but includes patients with the best overall response of stable disease. Comparisons of ORR and DCR were performed using the Chi‐square test. Differences in progression‐free survival (PFS) and overall survival (OS) were assessed with the log‐rank test and hazard ratios (HR) from Cox proportional hazard models, and median values were estimated with Kaplan–Meier analysis. Based on prior research performed by the Friends of Cancer Research, which identified 50% as the optimal cutoff for distinguishing response from non‐response in patients with non‐small cell lung cancer (NSCLC) who were treated with immune checkpoint inhibitors, a pre‐specified cutoff of 50% was used to define molecular response for all primary analyses [26]. Because that study was limited to NSCLC and this is a pan‐tumor cohort, cutoffs of 90% reduction and 100% reduction (complete clearance) were assessed in exploratory analysis. 100% reduction is aligned with several prior studies, while 90% was chosen to select patients with significant ctDNA reduction while being less stringent compared to 100% reduction [27, 28]. For any analysis leveraging ctDNA measurements from the C3D1 timepoint, PFS and OS analyses were landmarked from C3D1, and patients with progression/death before the landmark date were excluded. Correlation between ctDNA TF and SLD by RECIST 1.1, as well as ctDNA TF and maxVAF of predicted somatic and CH variants, was assessed with Pearson's correlation coefficient (*R*) and correlation between bTMB and tTMB was assessed with Lin's concordance correlation coefficient (CCC). Statistics, computation, and plotting were carried out using R 4.2.1 (Posit, RRID:SCR_001905) packages ggplot2 (RRID:SCR_014601), survminer (RRID:SCR_021094), survival (RRID:SCR_021137), epiR (RRID:SCR_021673),[ and tidyverse (RRID:SCR_019186).

## 3 Results

### 3.1 Study cohort characteristics

**Fig. 1:**
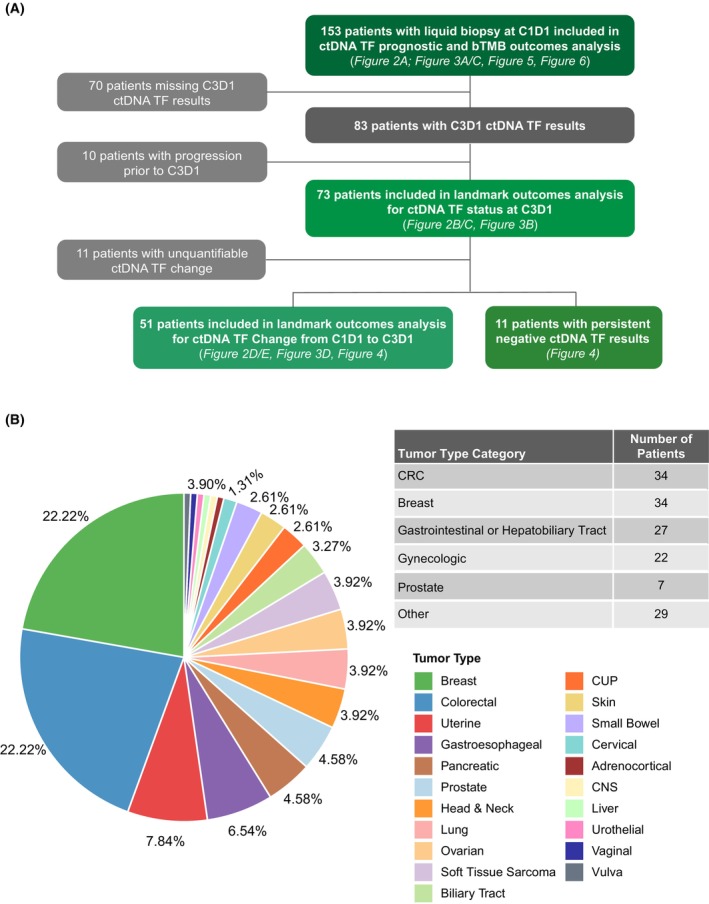
Study Cohort: Patients treated in the atezolizumab arm of the MyPathway basket trial (NCT02091141) were eligible for inclusion in this study. (A) 153 patients with ctDNA genomic profiling at C1D1 were included in pre‐treatment ctDNA TF and bTMB outcomes analysis. Patients missing ctDNA results at C3D1 or who progressed prior to C3D1 were excluded from outcomes analysis using on‐treatment ctDNA levels; an additional 22 patients were excluded from outcomes analysis focused on ctDNA change due to unquantifiable levels of change (11 with detectable ctDNA TF at both timepoints with estimates <2% and 11 patients with undetectable ctDNA at both timepoints). (B) MyPathway enrolled patients with a heterogeneous set of solid tumors. For subanalysis, patients were grouped into 5 primary tumor type groups with all other patients being classified as “other.” ctDNA, circulating tumor DNA; TF, tumor fraction; bTMB, blood tumor mutational burden; C1D1, cycle 1 day 1; C3D1, cycle 3 day 1; CRC, colorectal cancer; CUP, cancer of unknown primary; CNS, central nervous system.

In total, 153 patients who had successful ctDNA profiling results at baseline were included in this study (Fig. 1A). Colorectal cancers (CRC, 22%) and breast cancers (22%) were the most common tumor types and were analyzed separately, while all other tumor types were categorized as gastrointestinal/hepatobiliary (18%), gynecological (14%), prostate (5%), or other (19%) (Fig. 1B). As expected, clinical characteristics and genomics were variable across tumor types. Overall, this cohort was enriched for high tTMB tumors, consistent with trial eligibility criteria, with prostate and other tumor types having median tTMBs that were substantially higher than other diseases (Table S1). Additionally, ctDNA levels at C1D1 were variable by disease, with gynecological and prostate both having median levels of ctDNA TF >10% (15.0% and 18.0%, respectively; Fig. S1A). Predicted tumor‐derived genomic alterations detected in liquid biopsy at C1D1 were distinct among different tumor types and consistent with previous studies (Fig. S1B).

### 3.2 ctDNA tumor fraction and patient outcomes

**Fig. 2:**
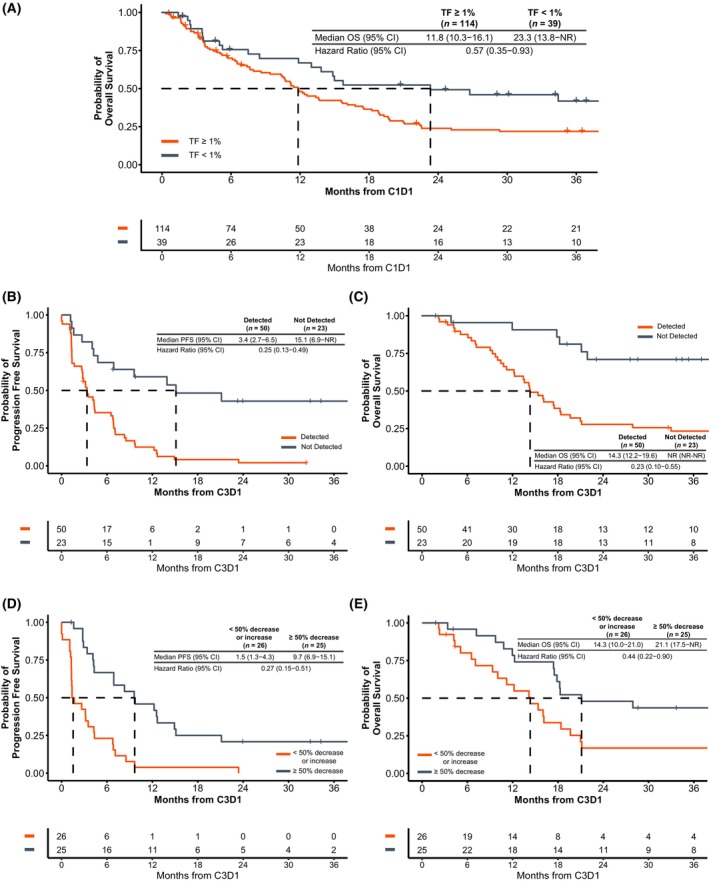
Correlation between outcomes and ctDNA detection and reduction. (A) Low ctDNA TF (<1%) at treatment initiation was associated with longer median OS. Lack of ctDNA detection at C3D1, irrespective of baseline ctDNA levels, was associated with prolonged (B) PFS and (C) OS from C3D1. Similarly to ctDNA detection, MR at C3D1 (defined as ≥50% decrease from C1D1) was associated with prolonged (D) PFS and (E) OS from C3D1. ctDNA, circulating tumor DNA; TF, tumor fraction; C1D1, cycle 1 day 1; C3D1, cycle 3 day 1; PFS, progression‐free survival; OS, overall survival; CI, confidence interval; NR, not reached.

In line with prior studies, ctDNA TF at C1D1 was strongly prognostic. Patients with low ctDNA TF (<1%; *n* = 39) had prolonged OS from the start of treatment (median 23.3 months) compared to those with high ctDNA TF (≥1%; 11.8 months; HR 0.57 [0.35–0.93]; Fig. 2A). No difference in confirmed ORR was observed (<1%: 18% vs 1%: 17%) affirming baseline ctDNA TF as a prognostic marker (Table S2).

We next assessed the association between on‐treatment ctDNA detection and patient outcomes irrespective of ctDNA levels detected at baseline. Seventy patients were excluded who did not have ctDNA results at C3D1 and an additional 10 were excluded who had progression documented prior to C3D1 (Fig. 1A). Of those who were missing ctDNA results, 48/70 had ceased treatment prior to C3D1 due to progression or death, while another 14/70 were no longer receiving treatment (Table S3). In patients with progression prior to C3D1, ctDNA was detected in 97% (28/29) of patients with an available end‐of‐treatment liquid biopsy. For 73 eligible patients for whom a C3D1 sample was available, those with undetectable ctDNA at C3D1 had prolonged PFS (15.1 vs 3.4 months from C3D1; HR 0.25 [0.13–0.49]; Fig. 2B) and OS (median not reached vs 14.3 months from C3D1; HR: 0.23 [0.10–0.55]; Fig. 2C). To confirm these findings were not driven by patients with ctDNA TF <1% at C1D1, and therefore better prognosis, we performed the analysis on the subgroup of patients with ctDNA TF ≥1% at C1D1 (*n* = 48); both median PFS (15.1 vs 3.3 months from C3D1; HR 0.24 [0.10–0.59]; Fig. S2A) and median OS (21.1 vs 15.4 months from C3D1; HR 0.40 [0.14–1.14]; Fig. S2B) were significantly longer in patients where ctDNA was undetectable at C3D1. Unlike ctDNA TF at C1D1, ctDNA TF detection at C3D1 was associated with higher ORR (52% vs 20%; Table S4); however, a high DCR was still seen in patients with detectable on‐treatment ctDNA (80%), likely due to the exclusion of patients who progressed prior to C3D1 from this analysis.

**Fig. 3:**
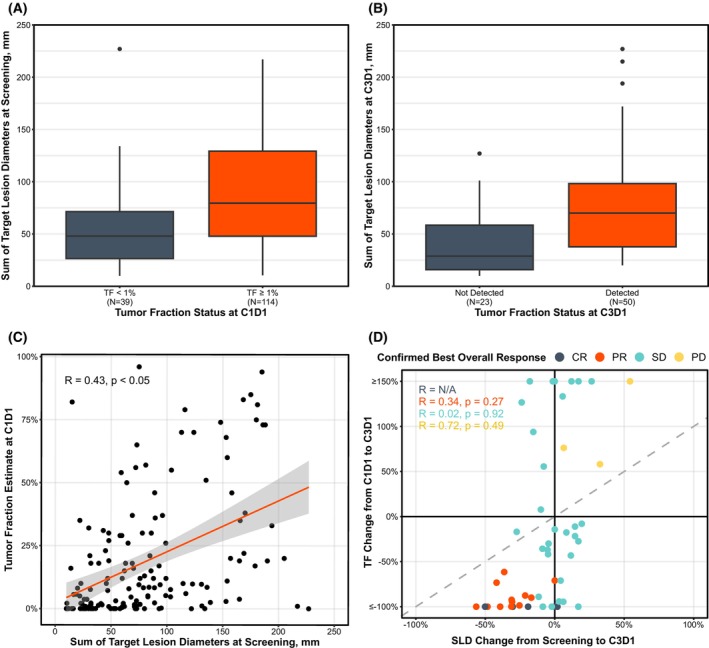
ctDNA detection is associated with SLD. (A) The SLD at baseline was significantly lower for patients with ctDNA not detected at C1D1 (error bars represent 1.5 times the IQR). (B) SLD at C3D1 was significantly lower for patients with ctDNA not detected at C3D1 (Error bars represent 1.5 times the IQR). (C) ctDNA TF and SLD had a modest positive correlation. Correlations were calculated with Pearson's correlation coefficient (error bars represent standard error). (D) All patients with PR or CR had a reduction or no change in SLD and had a reduction in ctDNA TF Correlations were calculated with Pearson's correlation coefficient. SLD, sum of target lesion diameters; IQR, interquartile range; TF, tumor fraction; C1D1, cycle 1 day 1; C3D1, cycle 3 day 1; CR, complete response; PR, partial response; SD, stable disease; PD, progressive disease.

Next, we grouped patients by molecular response (MR), defined as ≥50% decrease in ctDNA from C1D1 to C3D1. In patients with progression prior to C3D1, ctDNA increase of any magnitude was observed in 66% (21/32) of patients with an evaluable EOT liquid biopsy and only two patients (6%) achieved molecular response (Fig. S3). Twenty‐two additional patients were excluded due to unquantifiable change in ctDNA (11 below limit of quantification [2% at C1D1 and C3D1] and 11 with persistent negative ctDNA TF; Table S5). Patients who achieved MR (n = 25) had prolonged PFS (9.7 vs 1.5 months from C3D1; HR 0.27 [0.15–0.51]; Fig. 2D) and OS (21.1 vs 14.3 months from C3D1; HR: 0.44 [0.22–0.90]; Fig. 2E). Similar to ctDNA detection, MR was associated with ORR, with only patients who achieved MR also achieving a RECIST 1.1 response (68% vs 0%; Table S6). When using ≥90% decrease and 100% decrease as cutoffs for MR, similar results were observed for PFS and OS (Fig. S4). For patients who had undetectable ctDNA at C1D1 and C3D1 (persistent negative, n = 11), PFS was numerically longer when compared to patients with MR (13.9 vs 9.7 months; HR: 0.79 [0.31–1.97], Fig. S5A). Median overall survival in these patients was not reached, with 7/11 patients surviving for over 2 years and remaining on treatment at the time of data cutoff (Fig. S5B; Fig. 3). For patients with a 3^rd^ ctDNA assessment within 30 days of progression, 12/19 (63%) had ctDNA TF increase of any magnitude from their C3D1 ctDNA assessment (Fig. S6).

### 3.3 ctDNA tumor fraction, target lesion size, and RECIST 1.1

To better understand the relationship between ctDNA TF and imaging and RECIST 1.1, we analyzed the sum of (target) lesions longest diameter (SLD) as a proxy for tumor burden at both pre‐treatment and on‐treatment timepoints. Mirroring the C1D1 prognostic overall survival analysis, we compared SLD between patients with low vs high ctDNA TF. Patients with low ctDNA TF had lower median SLD compared to patients with high ctDNA TF (48.0 mm vs 79.5 mm; Fig. 3A). Similarly, when ctDNA TF was not detected at C3D1, median SLD was lower than in patients with detectable ctDNA (28.8 mm vs 70.0 mm; Fig. 3B). While median SLD was lower in patients with low ctDNA, there was only a weak continuous correlation between ctDNA TF at C1D1 and SLD at the time of screening (R = 0.43; Fig. 3C) and several patients were outliers. When comparing SLD by ctDNA TF levels within distinct tumor types, the trend of lower SLD in patients with low ctDNA persisted, suggesting tumor type was not the underlying reason for discrepancies between SLD and ctDNA TF (Fig. S7).

**Fig. 4:**
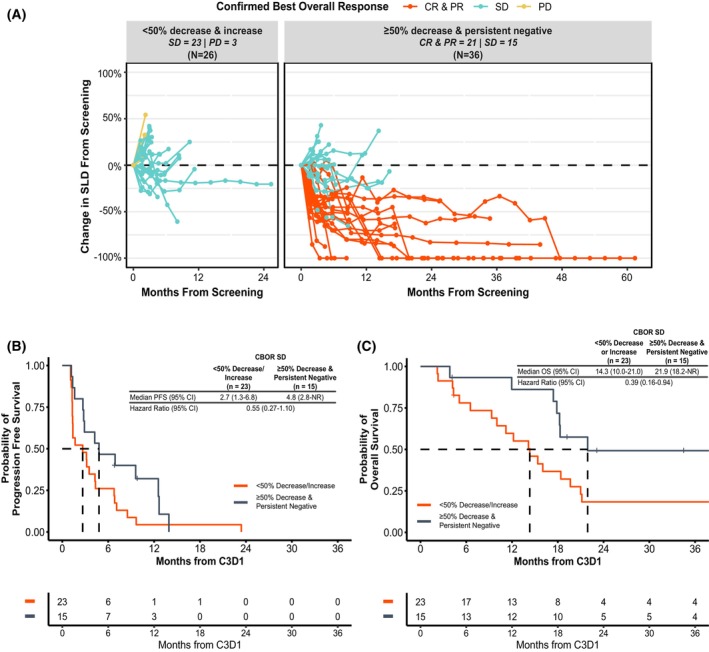
ctDNA change provides additional value beyond SLD or radiographic imaging. (A) Among the 21 patients with cBOR of CR or PR, all had a ctDNA decrease of at least 50% or were persistently negative for ctDNA, and all had a trend toward a reduction in SLD from screening. Among the 38 patients with SD as cBOR, no clear trend emerged among SLD or ctDNA response. Within patients with best confirmed response of SD, (B) PFS and (C) OS were both numerically longer in patients with ctDNA decrease or no change but the differences were not statistically significant. SLD, sum of target lesion diameters; C1D1, cycle 1 day 1; C3D1, cycle 3 day 1; PFS, progression‐free survival; OS, overall survival cBOR, confirmed best overall response; CR, complete response; PR, partial response; SD, stable disease; PD, progressive disease.

We assessed percent change in SLD from screening to C3D1 and compared it to percent change in ctDNA TF. Patients with a confirmed best overall response (cBOR by RECIST 1.1, *i.e*., consecutive radiographic results of the same or better response) of CR or PR all showed >50% ctDNA decrease by C3D1 despite only 2 having achieved a 50% reduction in SLD, though none had an increase in SLD (Fig. 3D). For patients with SD or PD, ctDNA TF changes were highly variable and showed no association with percent change in SLD at C3D1 or over the course of treatment (Figs 3D, 4A). However, additional analysis of patients with SD by RECIST 1.1 showed that patients with MR or persistent negative ctDNA TF have prolonged PFS (4.8 vs 2.7 months; HR = 0.55 [0.27–1.10]; Fig. 4B) and OS (21.9 vs 14.3 months; HR = 0.39 [0.16–0.94]; Fig. 4C) compared to those with <50% decrease or increase in ctDNA TF.

### 3.4 Blood tumor mutational burden and patient outcomes

**Fig. 5:**
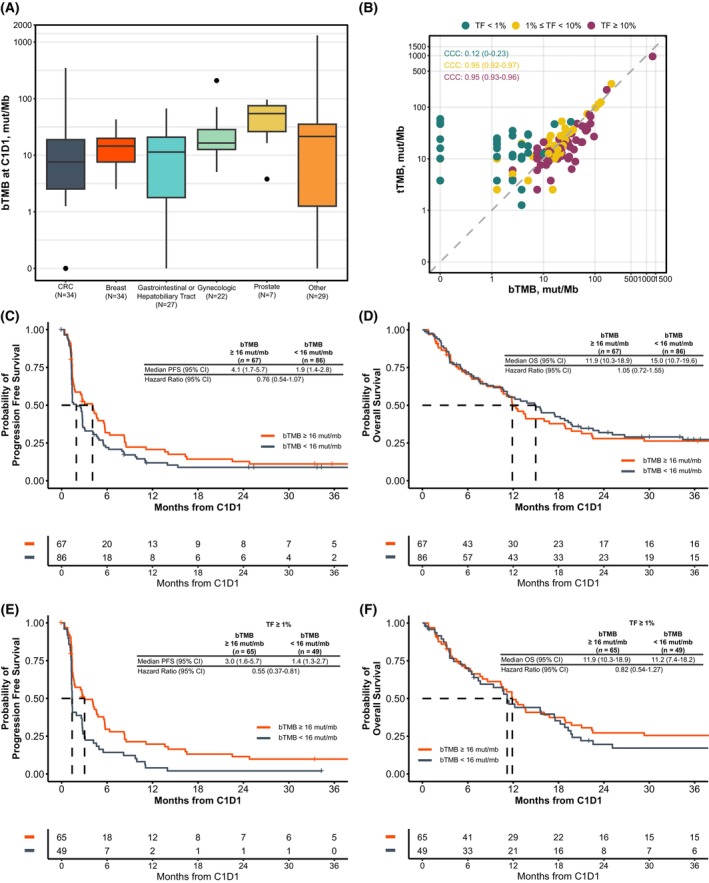
bTMB distribution, ctDNA TF, and association with outcomes. (A) bTMB at C1D1 by disease group. (B) When TF was 1–10% or ≥ 10%, there was a strong correlation between bTMB and tTMB. At TF <1%, there is no significant correlation between bTMB and tTMB, largely driven by a number of cases where bTMB is calculated at 0 mut/Mb, likely in samples with undetectable ctDNA. Correlation was calculated using Lin's concordance correlation coefficient. (C) PFS and (D) OS were not associated with elevated bTMB using the cutoff of 16 mut/Mb established for tTMB in previous work. (E) When limited to patients with ctDNA TF ≥1%, bTMB ≥16 mut/Mb was associated with decreased risk of progression. (F) This difference was not seen in OS. C1D1, cycle 1 day 1; bTMB, blood tumor mutational burden; mut/Mb, mutations per megabase; CRC, colorectal cancer; TF, tumor fraction; tTMB, tissue tumor mutational burden; PFS, progression‐free survival; OS, overall survival; CCC, Lin's concordance correlation coefficient.

Given the efficacy of atezolizumab in this cohort for patients whose tumors had tTMB ≥16 mut/Mb, we next explored bTMB and patient outcomes. bTMB was variable by tumor type (Fig. 5A) and the median bTMB in the full cohort was 12.6 mut/Mb, reflective of a population who were selected for enrollment based on elevated levels of tTMB. A strong correlation was observed between bTMB and tTMB at sufficient levels of ctDNA TF (both ≥10% and 1–10%: CCC = 0.95; Fig. 5B) but not in patients with low ctDNA TF (<1%: CCC = 0.12; Fig. 5B) where several patients had an estimated bTMB of 0 mut/Mb despite having elevated tTMB. Time between tissue and plasma collection was variable (median 284 days, interquartile range [IQR] 123–561 days, Fig. S8A) and did not impact the correlation between tTMB and bTMB (Fig. S8B). Additionally, MSI‐H concordance between tissue and liquid was high in patients with ctDNA TF ≥1% (12/14, 86%), while only one patient with ctDNA TF <1% whose tissue biopsy was MSI‐H had MSI‐H detected in their liquid biopsy (1/4, 25%).

Using a cutoff of ≥16 mut/Mb, elevated bTMB was associated with longer median PFS but the trend was not statistically significant (4.1 vs 1.9 months; HR: 0.76 [0.54–1.07]; Fig. 5C). No association with longer overall survival was observed (11.9 vs 15.0 months; HR: 1.05 [0.72–1.55]; Fig. 5D). Given the limited correlation between bTMB and tTMB in samples with low ctDNA TF, we then limited our analysis to patients with high ctDNA TF. Improved PFS (3.0 vs 1.4 months; HR: 0.55 [0.37–0.81], Fig. 5E) and higher ORR (27% v 4%, Table S7) were seen in patients with elevated bTMB but this did not translate to significantly longer OS (11.9 vs 11.2 months; HR: 0.82 [0.54–1.27], Fig. 5F).

In patients with low ctDNA TF, elevated tTMB (≥16 mut/Mb) remained predictive of both prolonged PFS (5.8 vs 2.6 months; HR: 0.42 [0.19–0.93]; Fig. S9A) and prolonged OS (median not reached vs 13.8 months; HR: 0.35 [0.13–0.95]; Fig. S9B). Using a higher cutoff of 25 mut/Mb, we observed a meaningful improvement in PFS for patients with elevated bTMB in the full population (4.2 vs 2.5 months; HR: 0.68 [0.46–1.00]; Fig. S9C) but no association with longer OS (11.8 vs 13.8 months; HR: 0.96 [0.63–1.49]; Fig. S9D).

### 3.5 Clonal hematopoiesis, ctDNA TF, ,and bTMB

**Fig. 6:**
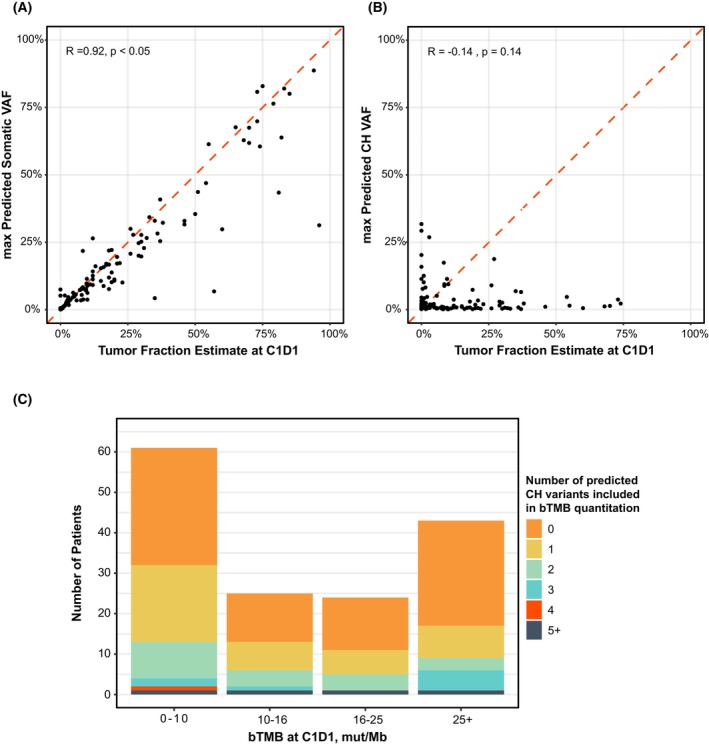
CH has minimal impact on ctDNA TF and bTMB estimations. Variants were algorithmically assigned predictions of origin. (A) For predicted tumor‐derived variants, the maxVAF at C1D1 was strongly associated with ctDNA TF while (B) no association was seen between ctDNA TF and the maxVAF for predicted CH variants. Correlation was calculated with Pearson's correlation coefficient (C) Predicted CH variants had a low impact on bTMB estimates. The majority of cases did not include CH, while estimates that included 2 or more CH variants were rare. CH, clonal hematopoiesis; bTMB, blood tumor mutational burden; mut/Mb, mutations per megabase; maxVAF, maximum variant allele frequency.

Because CH variants can confound ctDNA quantification and bTMB assessments, we analyzed the relationship between CH detection and both ctDNA TF and bTMB. When all variants, including germline, were used to calculate maxVAF, a moderate association was seen between ctDNA TF and maxVAF (*R* = 0.68; Fig. S10A), while removing just germline variants resulted in a stronger association between maxVAF and ctDNA TF (*R* = 0.90; Fig. S10B). Next, we compared the maxVAF for predicted tumor‐derived and predicted CH variants to ctDNA TF. As expected, a strong positive correlation between maxVAF for predicted tumor‐derived variants (*R* = 0.92; Fig. 6A) was observed, while no relationship between maxVAF for predicted CH variants and ctDNA TF was seen (*R* = −0.14 Fig. 6B). Studies have suggested that CH is associated with poor response to ICI [29, 30]. We found that patients with no detectable CH variants at C1D1 had significantly higher levels of ctDNA TF (median: 17.5% vs 4.6%; Fig. S11) confounding our ability to compare outcomes between patients with and without CH detected.

To understand the impact of CH on bTMB assessments, we applied VOP to all variants included in C1D1 bTMB calculations. Overall, 95% (3629/3814) of eligible variants were predicted to be tumor‐derived, 4% (146/3814) were predicted to be CH, and 1% (39/3814) were predicted to be germline. Impact on a patient level was minimal, with only 32/153 patients (21%) having two or more CH variants included in bTMB calculations (Fig. 6C). The impact on bTMB values was minimal, with a median change in bTMB of 2.53 mut/Mb after excluding CH, and many of these changes occurred in samples with bTMB <16 mut/Mb (Fig. 6C).

## 4 Discussion

ICIs have received FDA approvals for many solid tumor indications, along with companion biomarkers such as PD‐L1 expression and tTMB. However, these biomarkers are imperfect and there remains a need for improved patient outcomes. Furthermore, imaging for patients treated with ICIs can be ambiguous and additional strategies are needed to further stratify these patients and enhance treatment decision‐making. While imaging remains the gold standard both in routine clinical practice and in clinical trials, ctDNA dynamics have emerged as a promising biomarker with a growing body of evidence supporting its utility for assessing treatment.

Here, we studied the utility of plasma‐based biomarkers, including ctDNA TF, in solid tumor patients who were treated with atezolizumab as part of the MyPathway trial. As previously described [31, 32, 33, 34], we confirmed the validity of ctDNA quantification as a strong prognostic marker; patients with low ctDNA TF (<1%) had considerably longer OS compared to their high ctDNA TF counterparts (Fig. 2A). Additionally, we demonstrated the validity of on‐treatment ctDNA TF detection and MR (≥50% decrease in ctDNA TF from C1D1 to C3D1) for selecting patients with prolonged PFS and OS and high ORR (Fig. 2B,C). These findings align with previously published work showing an association between ctDNA decrease and/or clearance and improved PFS and OS for patients treated with immunotherapy [35, 36, 37, 38]. We acknowledge that other timepoints were not tested and may have a superior or similar association with outcomes, and investigation of other timepoints for ctDNA assessment is warranted [39]. Additionally, a large proportion of patients in this study progressed prior to C3D1, suggesting ctDNA assessment may need to take place earlier (*e.g.*, cycle 2 day 1) to add value to routine imaging and aid in treatment decisions. The inclusion of a wide variety of solid tumor types in this study shows broad potential applicability, though assessment of each individual tumor type with large, dedicated cohorts may provide a variety of optimal timepoints and situation‐specific definitions of MR.

Notably, 63% of patients treated past C3D1 with available ctDNA results exhibited an increase in ctDNA TF from C3D1 to EOT (Fig. S6) and 66% of patients who progressed prior to C3D1 had ctDNA TF increase in their end of treatment liquid biopsy (Fig. S3). Many studies of early‐stage cancers have sought to predict cancer recurrence prior to radiographic imaging by detection of ctDNA by various exquisitely sensitive methods [40, 41]. Our findings suggest ctDNA TF increase could be used to identify patients with a high probability of progression, but additional studies are needed to understand the timing of ctDNA increase in relation to radiographic progression.

RECIST 1.1 response was highly associated with on‐treatment ctDNA detection and MR. While ORR was notably higher for patients with ctDNA TF not detected and MR, DCR in the ctDNA TF detected and non‐MR patients was still higher than previously reported, possibly due to patients with rapid progression not receiving ctDNA testing at C3D1 or the use of RECIST 1.1 rather than iRECIST [42]. All patients with cBOR of CR or PR achieved MR, but ctDNA dynamics were variable for patients with SD or PD (Figs 3 and 4). Perhaps the use of the iRECIST criteria, which does not denote PD at the first detection of increase of ≥20% of the SLD in non‐target lesions (and requires confirmation of PD at a minimum 4 weeks) or at the appearance of new lesions, may correlate better with ctDNA change in patients who do not achieve a response by RECIST 1.1 criteria.

bTMB represents another potential plasma biomarker for predicting response to ICI. In the full treatment population, bTMB was not an effective biomarker for predicting patient outcomes, consistent with some other studies that have demonstrated limited utility of bTMB [43, 44]. However, concordance between tTMB and bTMB was directly associated with levels of ctDNA TF, suggesting that low levels of ctDNA in some patients likely biased these results. In patients with high ctDNA TF, prolonged PFS and improved ORR were observed in patients with elevated bTMB (≥16 mut/Mb; Fig. S9 and Table S7). An association of high bTMB with longer OS was not observed, potentially due to poorer prognosis overall in patients with high ctDNA TF. Other studies that have shown the validity of bTMB have similarly observed utility in a subset of patients with elevated levels of ctDNA [15, 16, 17].

The relationship between ctDNA TF and bTMB creates challenges in utilizing bTMB for clinical care. bTMB may be best suited for enrollment and exploratory biomarker analysis in early‐phase clinical trials. Here, endpoints such as response and PFS can help inform trial design for phase III randomized studies where OS remains the gold standard. For physicians receiving bTMB results with liquid biopsy profiling, results when ctDNA TF is low are largely unreliable. When ctDNA TF levels are sufficient (≥1%), elevated bTMB is strongly correlated with tTMB and could predict response to ICI, but given the prognostic implications of high ctDNA, combination therapies and/or more frequent follow‐up are warranted.

This study has several limitations. First, this trial enrolled patients who were heavily pre‐treated and had a heterogenous group of tumor types. While ORR within subgroups was generally similar to trends observed in the full cohort, sub‐analyses were underpowered, and further studies may be needed to establish the utility of ctDNA dynamics and bTMB in some of these tumor types. Additionally, the use of ctDNA monitoring and bTMB relies on a minimum quantity of ctDNA at treatment initiation. Earlier line patients may have differences in levels of ctDNA, and additional studies are needed to understand ctDNA detection across lines of therapy. Second, patients with rapid progression (<6 weeks) were excluded from time‐to‐event analysis. While we show that ctDNA increased in most patients with evaluable liquid biopsy near the time of progression, whether or not more time on therapy or an additional cycle of atezolizumab may have resulted in ctDNA decrease is unclear. Additional ctDNA timepoints would help clarify any subsequent changes. Finally, while the VOP algorithm is not directly incorporated into the TF algorithm, some inputs, such as fragmentomic signal, are the same. Therefore, assessing the impact of CH on ctDNA TF may be biased by the relationship between these two algorithms. Studies which include buffy coat sequencing as truth are ongoing and will be published in the future.

## 5 Conclusions

Tissue‐agnostic ctDNA monitoring is a viable tool for tracking patient response to ICIs such as atezolizumab. Both on‐treatment ctDNA TF detection and MR are strongly associated with patient outcomes. While tTMB is an effective biomarker for stratifying patients in this study, the dependence of bTMB on minimum levels of ctDNA TF (≥1%) limits the utility of bTMB.

## Conflict of interest

C.Sw. acknowledges grants from AstraZeneca, Boehringer Ingelheim, Bristol Myers Squibb, Pfizer, Roche‐Ventana, Invitae (previously Archer Dx – collaboration in minimal residual disease sequencing technologies), Ono Pharmaceutical, and Personalis. C.Sw. is the chief investigator for the AZ MeRmaiD 1 and 2 clinical trials and is the steering committee chair. C.Sw. is also the co‐chief investigator of the NHS Galleri trial financed by GRAIL and a paid member of GRAIL's scientific advisory board (SAB). C.Sw. receives consultant fees from Achilles Therapeutics (also a SAB member), Bicycle Therapeutics (also a SAB member), Genentech, Medicxi, the China Innovation Centre of Roche (formerly the Roche Innovation Centre – Shanghai), Metabomed (until July 2022) and the Sarah Cannon Research Institute. C.Sw. has received honoraria from Amgen, AstraZeneca, Bristol Myers Squibb, GlaxoSmithKline, Illumina, MSD, Novartis, Pfizer, and Roche‐Ventana. C.Sw. has previously held stock options in Apogen Biotechnologies and GRAIL and currently has stock options in Epic Bioscience and Bicycle Therapeutics, and has stock options and is co‐founder of Achilles Therapeutics. C.Sw. declares patent applications on methods to detect lung cancer (PCT/US2017/028013), targeting neoantigens (PCT/EP2016/059401), identifying patient response to immune checkpoint blockade (PCT/EP2016/071471), determining HLA loss of heterozygosity (PCT/GB2018/052004), predicting survival rates of patients with cancer (PCT/GB2020/050221), identifying patients who respond to cancer treatment (PCT/GB2018/051912) and methods for lung cancer detection (US20190106751A1). C.Sw. is an inventor on a European patent application (PCT/GB2017/053289) relating to assay technology to detect tumor recurrence. This patent has been licensed to a commercial entity, and under their terms of employment, C.Sw. is due a revenue share of any revenue generated from such license(s). RWM, CFBT, HT, JH, LWP, AG are employees of Foundation Medicine, Inc., a wholly owned subsidiary of Roche, and own stock in Roche. FMB a consulting or advisory role with AbbVie, Aduro Biotech, Alkermes, AstraZeneca, Debiopharm Group, eFFECTOR Therapeutics, Genentech, IBM Watson Health, Immunomedics, Infinity Pharmaceutical, Inflection Biosciences, Kolon Life Science, OrigiMed, PACT Pharma, Roche, Samsung Bioepis, Seattle Genetics, Silverback Therapeutics, Tyra Biosciences, Xencor, Zentalis, and Zymeworks; honoraria from Chugai Pharmaceuticals; institutional research funding from Aileron Therapeutics, AstraZeneca, Bayer, Calithera Biosciences, Curis, CytomX Therapeutics, Daiichi Sankyo, Debiopharm Group, eFFECTOR Therapeutics, Genentech, Guardant Health, Klus Pharma, Novartis, PUMA Biotechnology, Taiho Oncology, Inc., and Takeda Pharmaceuticals outside of the submitted work. RK has received research funding from Boehringer Ingelheim, Debiopharm, Foundation Medicine, Genentech, Grifols, Guardant, Incyte, Konica Minolta, Medimmune, Merck Serono, Omniseq, Pfizer, Sequenom, Sysmex, Takeda, and TopAlliance and from the NCI; as well as consultant and/or speaker fees and/or advisory board/consultant for Actuate Therapeutics, AstraZeneca, Bicara Therapeutics, Inc., Biological Dynamics, Caris, Daiichi, Datar Cancer Genetics, EISAI, EMD Serono, EOM Pharmaceuticals, Iylon, Jackson Laboratories, LabCorp, Lanauria Therapeutics, Merck, NeoGenomics, Neomed, Pfizer, Precirix, Prosperdtx, Quanta Therapeutics, Recordati, Regeneron, Roche, TD2/Volastra, Turning Point Therapeutics, XBiotech; has an equity interest in CureMatch Inc.; serves on the Board of CureMatch and CureMetrix and XZOM; and is a co‐founder of CureMatch. CJS: Research Funding paid to institution by: Janssen, Astellas, Pfizer, Bayer; Consulting, or Advisory Role: Johnson and Johnson, Astellas, Bayer, Genentech/Roche, Pfizer, Lilly; CellCentric, PointBiopharma; Amphista, Astra Zeneca, Novartis, Advancell, QEDDI, BMS. Royalties and other Intellectual Property: Parthenolide (Indiana University); dimethylamino parthenolide (Leuchemix); Exelixis: Abiraterone plus cabozantinib combination; FRAS1 SNP and tristetraprolin as biomarkers of lethal prostate cancer; Stock or Other Ownership: Leuchemix. HAB: employment, leadership position, and stock and other ownership interests in HCA Healthcare; research funding from Abbvie, Agios, Archer, ARMO BioSciences, Array BioPharma, Arvinas, AstraZeneca, Bayer, BeiGene, BioAtla, BioMed Valley Discoveries, BioTheryX, Boehringer Ingelheim, Bristol Myers Squibb, CALGB, CicloMed, Coordination Pharmaceuticals, CytomX Therapeutics, eFFECTOR Therapeutics, EMD Serono, Foundation Medicine, Gilead Sciences, GlaxoSmithKline, Gossamer Bio, Harpoon therapeutics, Hengrui Therapeutics, Incyte, Janssen, Jounce Therapeutics, Kymab, Lilly, Macrogenics, MedImmune, Merck, Moderna Therapeutics, NGM Biopharmaceuticals, Novartis, Pfizer, Revolution Medicines, Roche/Genentech, Ryvu Therapeutics, Seattle Genetics, Takeda/Millennium, Tesaro, TG Therapeutics, Verastem, Vertex, XBiotech, Zymeworks (Inst); uncompensated relationships – AstraZeneca, Bayer, Boehringer Ingelheim, Bristol Myers Squibb, GRAIL, Incyte, Novartis, TG Therapeutics, Vincerx Pharma (Inst). DRS: institutional funding (AstraZeneca, Genentech/Roche, Novartis, Celgene, Bristol Myers Squibb, Pfizer, Boehringer Ingelheim, Abbvie, Foundation Medicine, GlaxoSmithKline, Lilly, Merck, Moderna Therapeutics, Nektar, Takeda, Amgen, University of Texas Southwestern Medical Center – Simmons Cancer Center, G1 Therapeutics, Neon Therapeutics, Celldex, Clovis Oncology, Daiichi Sankyo, EMD Serono, Acerta Pharma, Oncogenex, Astellas Pharma, GRAIL, Transgene, Aeglea Biotherapeutics, Tesaro, Ipsen, ARMO BioSciences, and Millennium); consultation (AstraZeneca, TRM Oncology, Precision Oncology, Evelo Therapeutics, Illumina, PharmaMar, Genentech/Roche, Novartis, Celgene, Bristol Myers Squibb, Pfizer, Boehringer Ingelheim, Abbvie, Foundation Medicine, GlaxoSmithKline, Lilly, and Merck); travel and expenses (AstraZeneca, Genzyme, Intuitive Surgical, Purdue Pharma, Spectrum Pharmaceuticals, Sysmex, EMD Serono, Genentech/Roche, Novartis, Celgene, Bristol Myers Squibb, Pfizer, Boehringer Ingelheim, Abbvie, Foundation Medicine, GlaxoSmithKline, Lilly, and Merck). JM, BY, TS, KS are employees of Genentech, Inc. and own stock in Roche. CSc is a contractor employed by Roche and was employed by Impact Clinical, LLC at the time of the MyPathway trial/data collection. CFF: Institutional research support from Marengo Therapeutics, Immunocore, Hotspot Therapeutic, Eli Lilly, Volastra Therapeutics, Merck, Bristol Myers Squibb, AstraZeneca, and Genentech. She reports personal fees from Eli Lilly, travel support from Puma Biotechnology, and Scientific Advisory Committee (uncompensated) participation for Genentech and Merck.

## Author contributions

CSw: conceptualization, investigation, resources, writing – review & editing. RWM: conceptualization, methodology, supervision, writing – original draft. CFBT: formal analysis, software, visualization, writing – review & editing. FMB: investigation, resources, writing – review & editing. CJS: investigation, resources, writing – review & editing. RK: investigation, resources, writing – review & editing. HAB: investigation, resources, writing – review & editing. DRS: investigation, resources, writing – review & editing. HT: conceptualization, writing – original draft. JH: methodology, software, writing – review & editing. JM: data curation, writing – review & editing. BY: data curation, writing – review & editing. TS: project administration, writing – review & editing. CSc: project administration, writing – review & editing. LWP: conceptualization, supervision, writing – original draft. AG: supervision, writing – review & editing. KS: conceptualization, funding acquisition, project administration, supervision, writing – review & editing. CFF: conceptualization, investigation, resources, writing – review & editing.

## Supporting information


**Fig. S1.** ctDNA TF and tumor‐derived alterations at C1D1 by tumor type. (A) Distribution of ctDNA TF at C1D1 by tumor type. Patients with detected but non‐quantifiable ctDNA were excluded from this figure. (B) Landscape of tumor‐derived alterations by tumor type. ctDNA, circulating tumor DNA; TF, tumor fraction; LOQ, limit of quantification; CRC, colorectal cancer; C1D1, cycle 1 day 1; ND, not detected.

**Fig. S2.** Outcomes in patients with high ctDNA TF (≥1%) at C1D1 based on ctDNA detection at C3D1. ctDNA TF detection at C3D1 was assessed for association with outcomes in patients with high ctDNA TF (≥1%) at C1D1 to ensure that findings from the full cohort were not primarily driven by patients with low ctDNA at treatment start. Like the full cohort, lack of ctDNA detection at C3D1 was associated with prolonged (B) PFS and (C) OS from C3D1. ctDNA, circulating tumor DNA; TF, tumor fraction; C1D1, cycle 1 day 1; C3D1, cycle 3 day 1; PFS, progression‐free survival; OS, overall survival; CI, confidence interval; NR, not reached.

**Fig. S3.** Patient outcomes by ICI predictive biomarkers and prognostic factors for patients who were exclude from time‐to‐event analysis due to progression prior to C3D1. For each patient, the tumor type, bTMB at C1D1, tTMB at C1D1, MSI status at C1D1, and ctDNA TF changes from C1D1 to EOT are shown. Additionally, the time on therapy is shown in horizontal bars at right, with known death and disease progression events marked. The majority of patients had ctDNA increase (21/32) at EOT while only two (6%) had molecular response. ctDNA, circulating tumor DNA; TF, tumor fraction; C1D1, cycle 1 day 1; C3D1, cycle 3 day 1; bTMB, blood tumor mutational burden; tTMB, tissue tumor mutational burden; MSI, microsatellite instability; PD, progressive disease; ND, not detected; EOT, end of treatment.

**Fig. S4.** Correlation between outcomes and ctDNA TF reduction at 90% and 100%. Near or full clearance at C3D1 (defined as ≥90% decrease from C1D1) was associated with prolonged (A) PFS and (B) OS from C3D1. Complete clearance at C3D1 (defined as 100% decrease from C1D1) was associated with prolonged (C) PFS and (D) OS from C3D1. ctDNA, circulating tumor DNA; TF, tumor fraction C3D1, cycle 3 day 1; PFS, progression‐free survival; OS, overall survival; CI, confidence interval; NR, not reached.

**Fig. S5.** PFS and OS for patients with persistent negative ctDNA TF status. (A, B) Patients who were ctDNA TF negative at both C1D1 and C3D1 (persistent negative) were not included in primary analysis of ctDNA TF change. Median PFS from C3D1 was longer for persistent negative patients and a strong prognostic signal was seen in OS for these patients. ctDNA, circulating tumor DNA; TF, tumor fraction; C1D1, cycle 1 day 1; C3D1, cycle 3 day 1; PFS, progression‐free survival; OS, overall survival; CI, confidence interval; NR, not reached.

**Fig. S6.** Patient outcomes by ICI predictive and prognostic factors. For each patient, the tumor type, bTMB at C1D1, tTMB at C1D1, MSI status at C1D1, and ctDNA TF changes from C1D1 to C3D1 are shown. Additionally, the time on therapy is shown in horizontal bars at right, with known death and disease progression events marked. Bars are color coded to cBOR. Additionally, ctDNA TF change from C3D1 to EOT. is shown in triangles. ctDNA, circulating tumor DNA; TF, tumor fraction; C1D1, cycle 1 day 1; C3D1, cycle 3 day 1; bTMB, blood tumor mutational burden; tTMB, tissue tumor mutational burden; MSI, microsatellite instability; cBOR, confirmed best overall response; ND, not detected; CR, complete response; PR, partial response; SD, stable disease; PD, progressive disease; EOT, end of treatment; CUP, cancer of unknown primary.

**Fig. S7.** ctDNA TF and SLD by disease group. (A) Stratified at ctDNA TF = 1% at C1D1. (B) Stratified by detected vs not detected at C3D1. ctDNA, circulating tumor DNA; TF, tumor fraction; SLD, sum of target lesion diameters; C1D1, cycle 1 day 1; C3D1, cycle 3 day 1; CRC, colorectal cancer.

**Fig. S8.** Correlation between bTMB and tTMB by time between sample collection. (A) Distribution of time between tissue and plasma collection for bTMB and tTMB correlation analysis. (B) bTMB and tTMB remained correlated when TF was >1%, irrespective of time between tissue and plasma collection. ctDNA, circulating tumor DNA; TF, tumor fraction; bTMB, blood tumor mutational burden; mut/Mb, mutations per megabase; tTMB, tissue tumor mutational burden; CCC, Lin's concordance correlation coefficient.

**Fig. S9.** Outcomes by tTMB status in patients with low ctDNA TF (<1%) and outcomes by bTMB status using a cutoff of 25 mut/Mb. (A, B) In patients with ctDNA TF <1%, where bTMB is not reliable, tTMB ≥16 mut/mb is strongly associated with decreased risk of progression and death. (C, D) In the full treatment cohort, a cutoff of 25 mut/mb was associated with improved PFS, but not OS, in all patients. C1D1, cycle 1 day 1; ctDNA, circulating tumor DNA; TF, tumor fraction; bTMB, blood tumor mutational burden; mut/Mb, mutations per megabase; tTMB, tissue tumor mutational burden; PFS, progression‐free survival; OS, overall survival.

**Fig. S10.** Correlation between maxVAF and ctDNA TF by alteration origin. (A) For all variants, the maximum VAF at C1D1 was moderately associated with ctDNA TF. (B) When predicted germline variants were excluded, a stronger association between maximum VAF at C1D1 and ctDNA TF was observed. C1D1, cycle 1 day 1; ctDNA, circulating tumor DNA; TF, tumor fraction; maxVAF, maximum variant allele frequency; CH, clonal hematopoiesis.

**Fig. S11.** ctDNA TF at C1D1 stratified by the detection of CH alterations. Patients with no predicted CH alterations had higher levels of ctDNA TF. ctDNA, circulating tumor DNA; TF, tumor fraction; CH, clonal hematopoiesis; C1D1, cycle 1 day 1; LOQ, limit of quantification; ND, not detected.

**Table S1.** Patient characteristics at start of treatment. TF, tumor fraction; C1D1, cycle 1 day 1; IQR, interquartile range; TMB, tumor mutational burden; bTMB, blood TMB, tTMB, tissue TMB; TPS, tumor proportion score.

**Table S2.** Best confirmed response (cBOR) based of ctDNA tumor fraction at cycle 1 day 1 (C1D1). CR, complete response; PR, partial response; SD, stable disease; PD, progressive disease; ORR, objective response rate (CR + PR); DCR, disease control rate (CR + PR + SD).

**Table S3.** Patient characteristics for patients who were excluded from outcomes analysis due to missing cycle 3 day 1 (C3D1) liquid biopsy or early progression compared to patients who were included. Response measured by RECIST 1.1. CR, complete response; PR, partial response; SD, stable disease; PD, progressive disease; IQR, interquartile range; TMB, tumor mutational burden; mut/mb, mutations per megabase; bTMB, blood TMB; tTMB, tissue TMB; TPS, tumor proportion score.

**Table S4.** Best confirmed response (cBOR) based on ctDNA tumor fraction detection at cycle 3 day 1 (C3D1). CR, complete response; PR, partial response; SD, stable disease; PD, progressive disease; ORR, objective response rate (CR + PR); DCR, disease control rate (CR + PR + SD).

**Table S5.** Patient characteristics for those with persistent negative or unquantifiable ctDNA at cycle 3 day 1 (C3D1). CR, complete response; PR, partial response; SD, stable disease; PD, progressive disease; TMB, tumor mutational burden; mut/mb, mutation per megabase; bTMB, blood TMB; tTMB, tissue TMB; TPS, tumor proportion score; IQR, interquartile.

**Table S6.** Best confirmed response based on change in ctDNA tumor fraction detection at cycle 3 day 1 (C3D1). CR, complete response; PR, partial response; SD, stable disease; PD, progressive disease; ORR, objective response rate (CR + PR); DCR, disease control rate (CR + PR + SD).

**Table S7.** Best confirmed response stratified by ctDNA tumor fraction (TF) and blood tumor mutational burden (bTMB). CR, complete response; PR, partial response; SD, stable disease; PD, progressive disease; ORR, objective response rate (CR + PR); DCR, disease control rate (CR + OR + SD); CI, confidence interval.

## Data Availability

All relevant data are provided within the article and its accompanying Supplementary Material. Because of Health Insurance Portability and Accountability Act requirements, we are not consented to share individualized patient genomic data, which contains potentially identifying or sensitive patient information. Foundation Medicine is committed to collaborative data analysis, and we have well‐established and widely used mechanisms by which investigators can query our core genomic database of >750 000 deidentified sequenced cancers to obtain aggregated datasets. More information and mechanisms for data access can be obtained by contacting the corresponding authors or the Foundation Medicine Data Governance Council at data.governance.council@foundationmedicine.com. For up‐to‐date details on Roche's Global Policy on the Sharing of Clinical Information and how to request access to related clinical study documents, see https://go.roche.com/data_sharing. Qualified researchers may request access to deidentified patient‐level data and clinical study documentation via the following link: https://www.roche.com/innovation/process/clinical‐trials/data‐sharing/request. Anonymized records for individual patients across more than one data source external to Roche cannot, and should not, be linked due to a potential increase in risk of patient reidentification.
