# Supplementary figures and images for: Circulating tumor DNA monitoring and blood tumor mutational burden in patients with metastatic solid tumors treated with atezolizumab
Source: Mol Oncol. 2025 May 28;19(11):3060–78. doi: 10.1002/1878-0261.70054 (PMC12591311; doi:10.1002/1878-0261.70054)

**A**

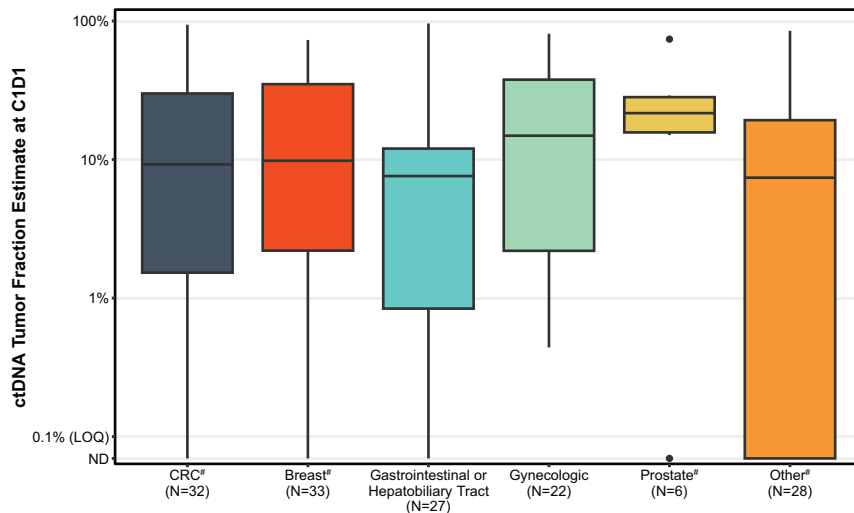

**B**

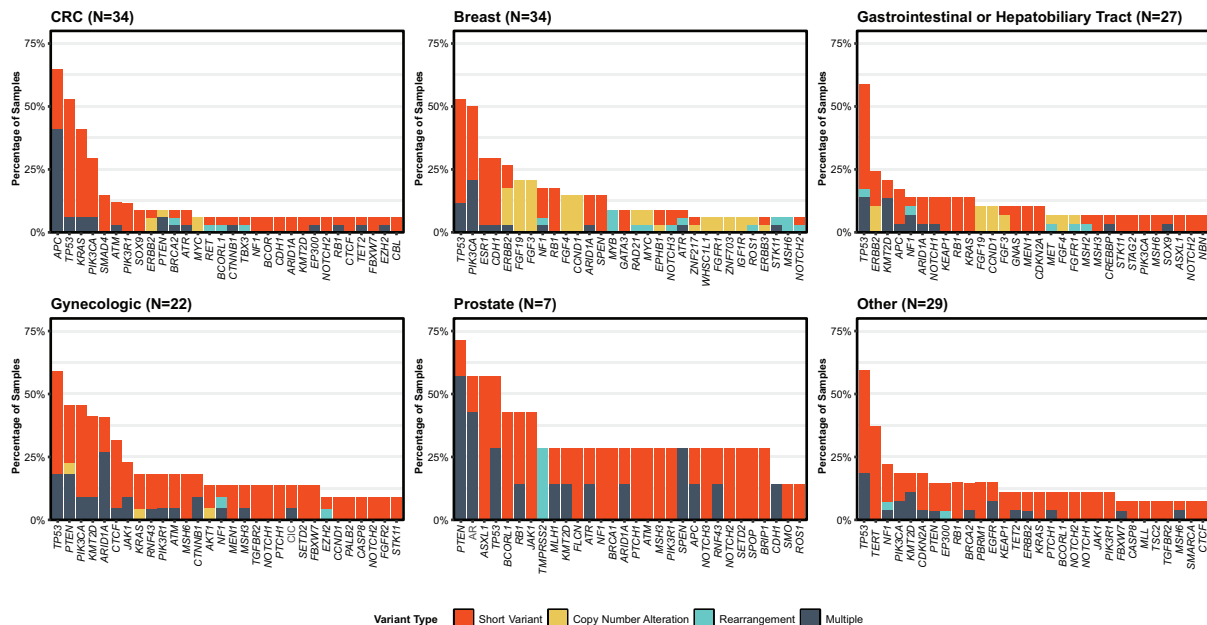

**Supplemental Figure 1**

Supplement: Supplementary file 1 — Fig. S1. ctDNA TF and tumor‐derived alterations at C1D1 by tumor type. (A) Distribution of ctDNA TF at C1D1 by tumor type. Patients with detected but non‐quantifiable ctDNA were excluded from this figure. (B) Landscape of tumor‐derived alterations by tumor type. ctDNA, circulating tumor DNA; TF, tumor fraction; LOQ, limit of quantification; CRC, colorectal cancer; C1D1, cycle 1 day 1; ND, not detected. [file MOL2-19-3060-s007.pdf]

**A**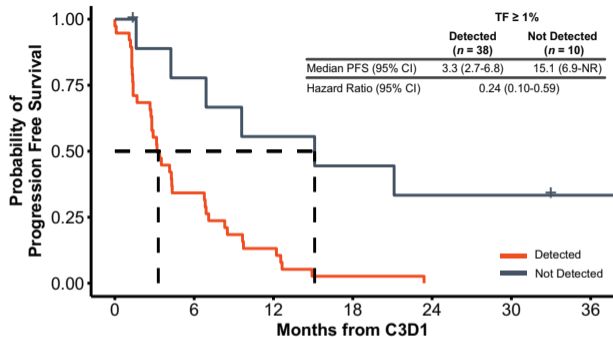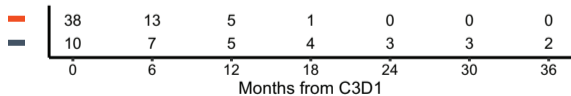**B**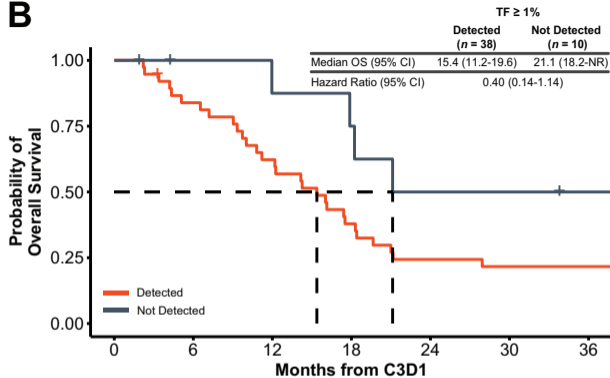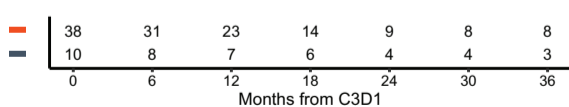**Supplemental Figure 2**

Supplement: Supplementary file 2 — Fig. S2. Outcomes in patients with high ctDNA TF (≥1%) at C1D1 based on ctDNA detection at C3D1. ctDNA TF detection at C3D1 was assessed for association with outcomes in patients with high ctDNA TF (≥1%) at C1D1 to ensure that findings from the full cohort were not primarily driven by patients with low ctDNA at treatment start. Like the full cohort, lack of ctDNA detection at C3D1 was associated with prolonged (B) PFS and (C) OS from C3D1. ctDNA, circulating tumor DNA; TF, tumor fraction; C1D1, cycle 1 day 1; C3D1, cycle 3 day 1; PFS, progression‐free survival; OS, overall survival; CI, confidence interval; NR, not reached. [file MOL2-19-3060-s017.pdf]

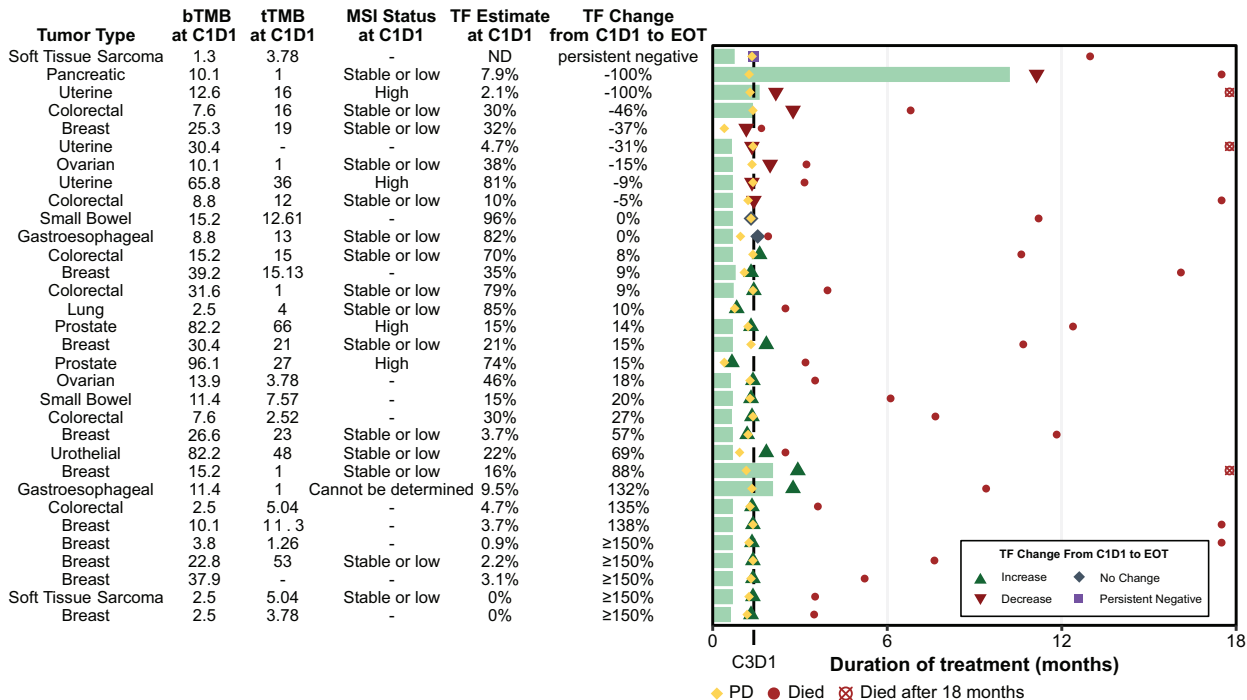

**Supplemental Figure 3**

Supplement: Supplementary file 3 — Fig. S3. Patient outcomes by ICI predictive biomarkers and prognostic factors for patients who were exclude from time‐to‐event analysis due to progression prior to C3D1. For each patient, the tumor type, bTMB at C1D1, tTMB at C1D1, MSI status at C1D1, and ctDNA TF changes from C1D1 to EOT are shown. Additionally, the time on therapy is shown in horizontal bars at right, with known death and disease progression events marked. The majority of patients had ctDNA increase (21/32) at EOT while only two (6%) had molecular response. ctDNA, circulating tumor DNA; TF, tumor fraction; C1D1, cycle 1 day 1; C3D1, cycle 3 day 1; bTMB, blood tumor mutational burden; tTMB, tissue tumor mutational burden; MSI, microsatellite instability; PD, progressive disease; ND, not detected; EOT, end of treatment. [file MOL2-19-3060-s014.pdf]

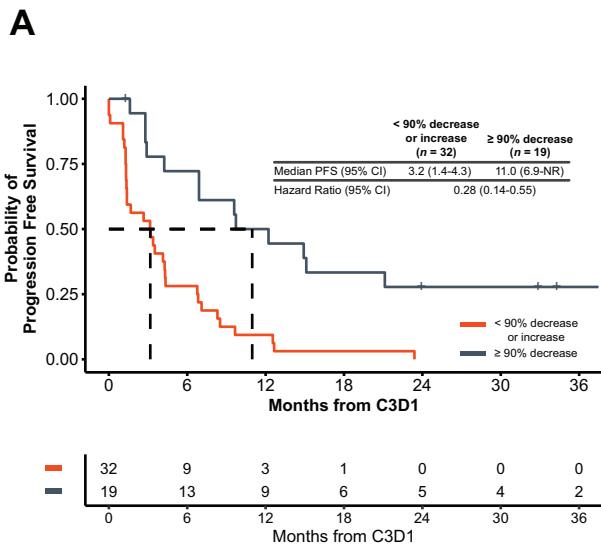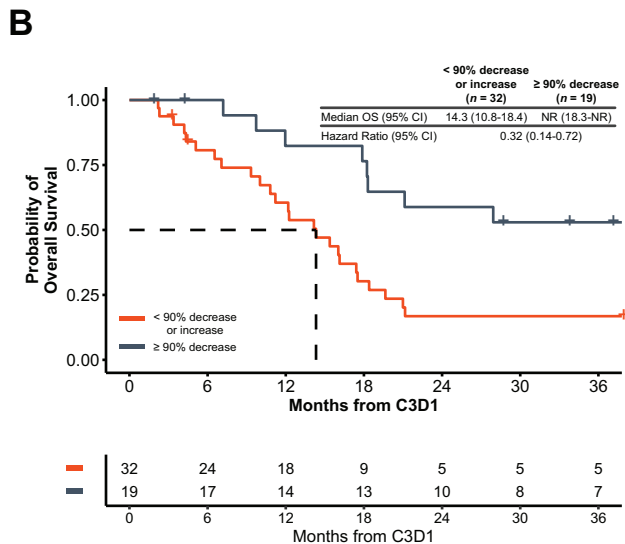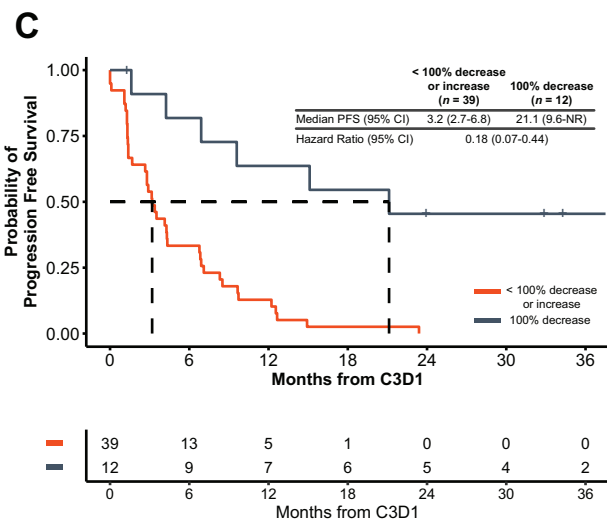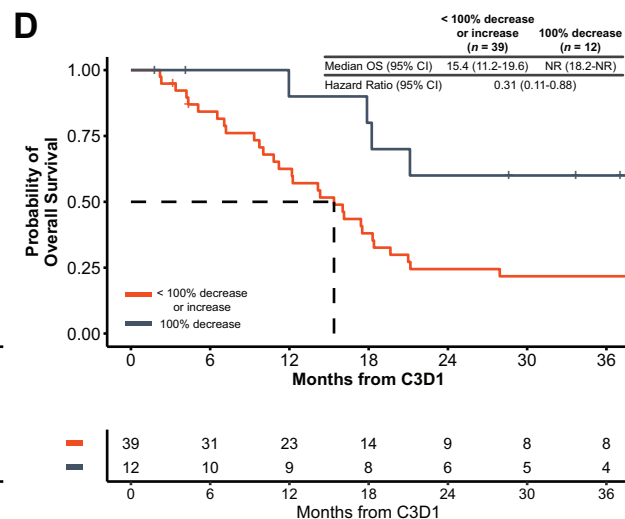

**Supplemental Figure 4**

Supplement: Supplementary file 4 — Fig. S4. Correlation between outcomes and ctDNA TF reduction at 90% and 100%. Near or full clearance at C3D1 (defined as ≥90% decrease from C1D1) was associated with prolonged (A) PFS and (B) OS from C3D1. Complete clearance at C3D1 (defined as 100% decrease from C1D1) was associated with prolonged (C) PFS and (D) OS from C3D1. ctDNA, circulating tumor DNA; TF, tumor fraction C3D1, cycle 3 day 1; PFS, progression‐free survival; OS, overall survival; CI, confidence interval; NR, not reached. [file MOL2-19-3060-s018.pdf]

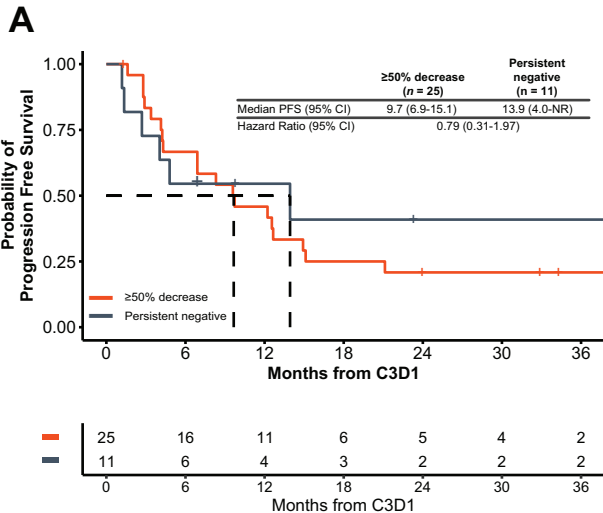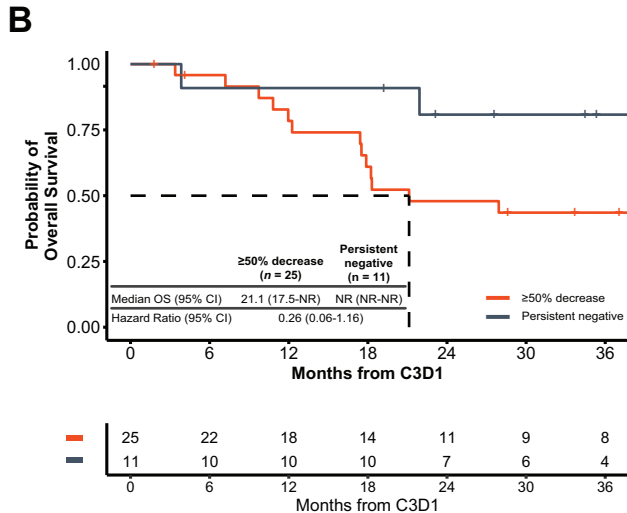

**Supplemental Figure 5**

Supplement: Supplementary file 5 — Fig. S5. PFS and OS for patients with persistent negative ctDNA TF status. (A, B) Patients who were ctDNA TF negative at both C1D1 and C3D1 (persistent negative) were not included in primary analysis of ctDNA TF change. Median PFS from C3D1 was longer for persistent negative patients and a strong prognostic signal was seen in OS for these patients. ctDNA, circulating tumor DNA; TF, tumor fraction; C1D1, cycle 1 day 1; C3D1, cycle 3 day 1; PFS, progression‐free survival; OS, overall survival; CI, confidence interval; NR, not reached. [file MOL2-19-3060-s005.pdf]

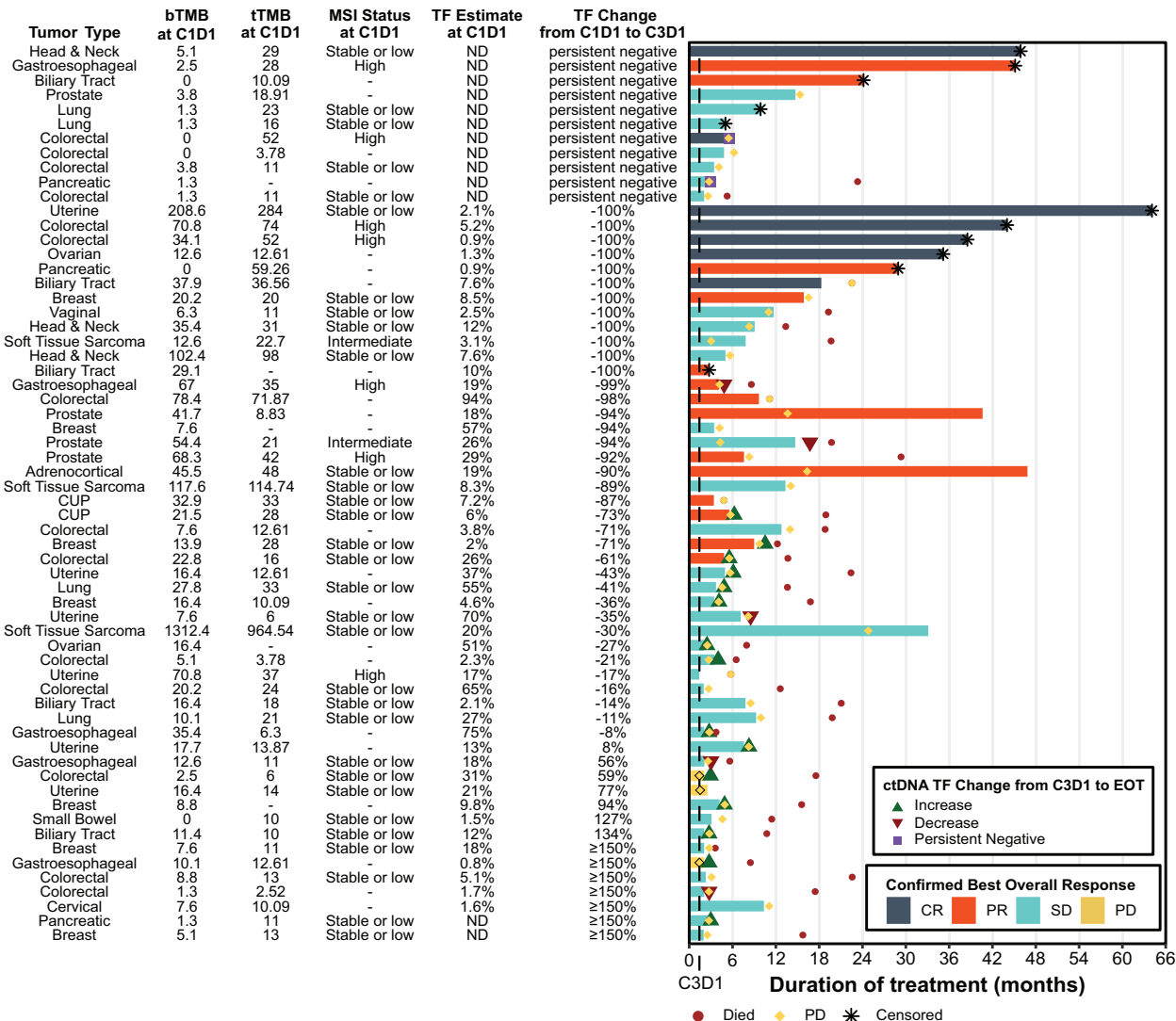

**Supplemental Figure 6**

Supplement: Supplementary file 6 — Fig. S6. Patient outcomes by ICI predictive and prognostic factors. For each patient, the tumor type, bTMB at C1D1, tTMB at C1D1, MSI status at C1D1, and ctDNA TF changes from C1D1 to C3D1 are shown. Additionally, the time on therapy is shown in horizontal bars at right, with known death and disease progression events marked. Bars are color coded to cBOR. Additionally, ctDNA TF change from C3D1 to EOT. is shown in triangles. ctDNA, circulating tumor DNA; TF, tumor fraction; C1D1, cycle 1 day 1; C3D1, cycle 3 day 1; bTMB, blood tumor mutational burden; tTMB, tissue tumor mutational burden; MSI, microsatellite instability; cBOR, confirmed best overall response; ND, not detected; CR, complete response; PR, partial response; SD, stable disease; PD, progressive disease; EOT, end of treatment; CUP, cancer of unknown primary. [file MOL2-19-3060-s004.pdf]

**A**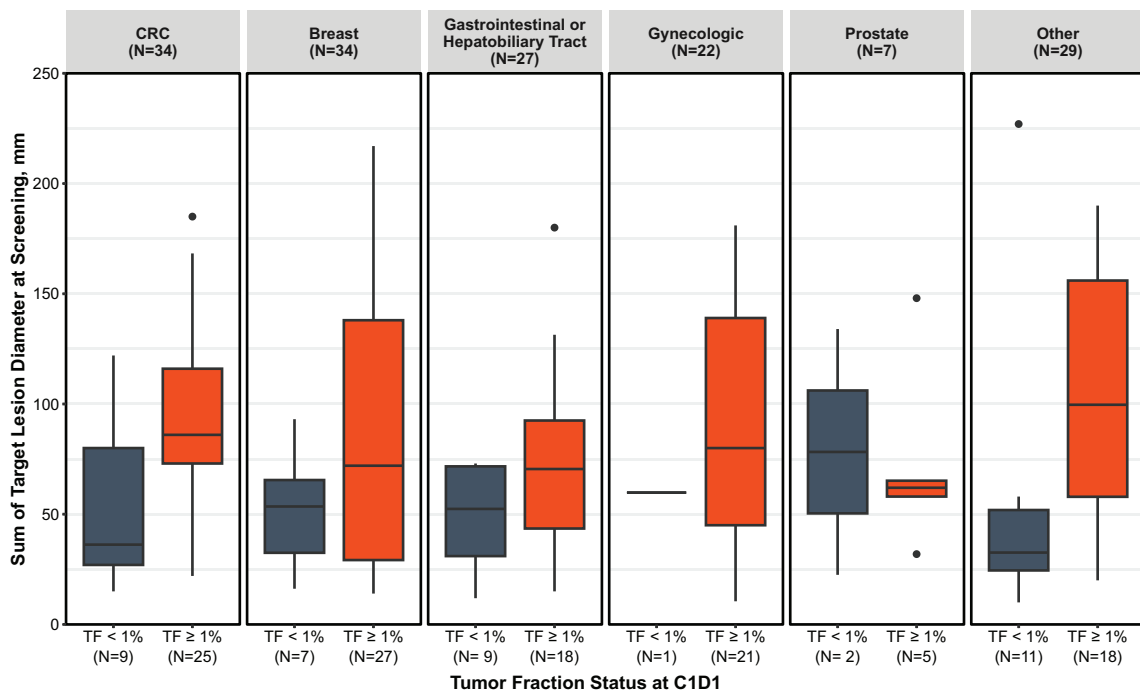**B**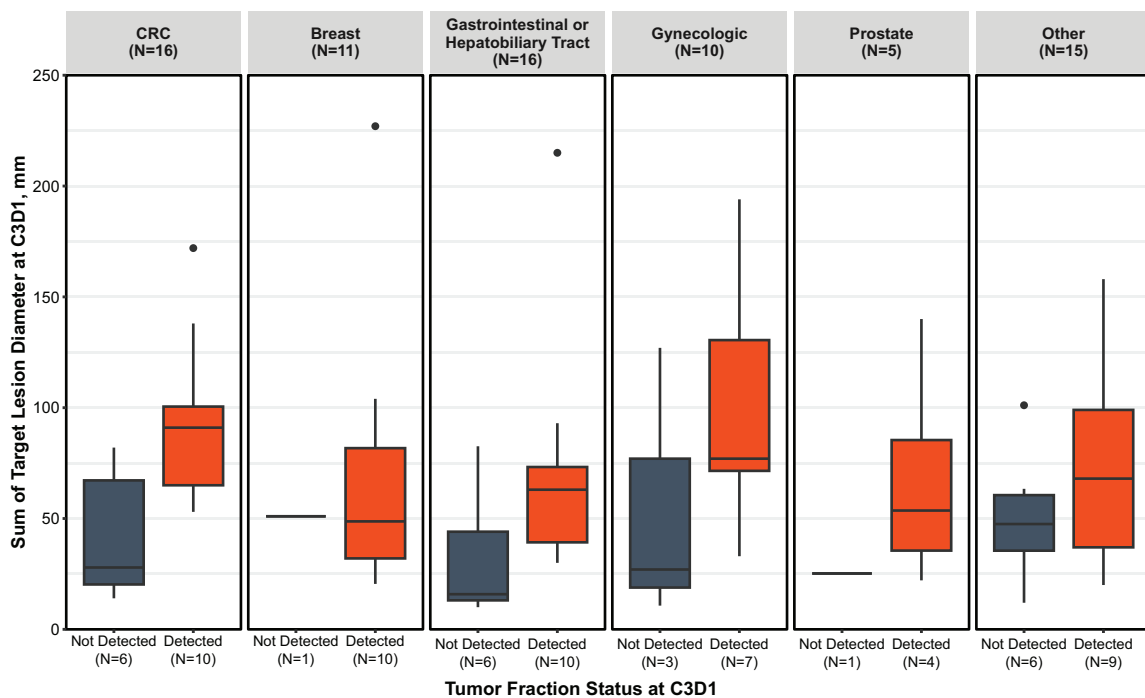**Supplemental Figure 7**

Supplement: Supplementary file 7 — Fig. S7. ctDNA TF and SLD by disease group. (A) Stratified at ctDNA TF = 1% at C1D1. (B) Stratified by detected vs not detected at C3D1. ctDNA, circulating tumor DNA; TF, tumor fraction; SLD, sum of target lesion diameters; C1D1, cycle 1 day 1; C3D1, cycle 3 day 1; CRC, colorectal cancer. [file MOL2-19-3060-s016.pdf]

**A**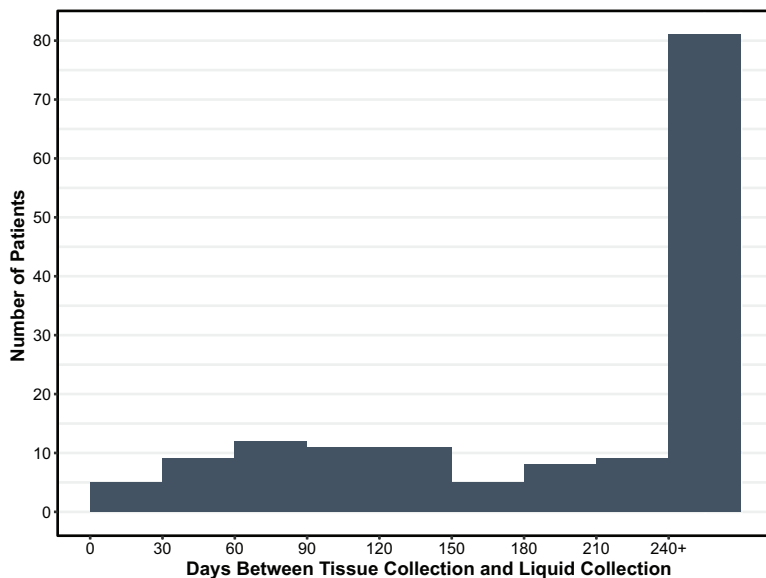**B**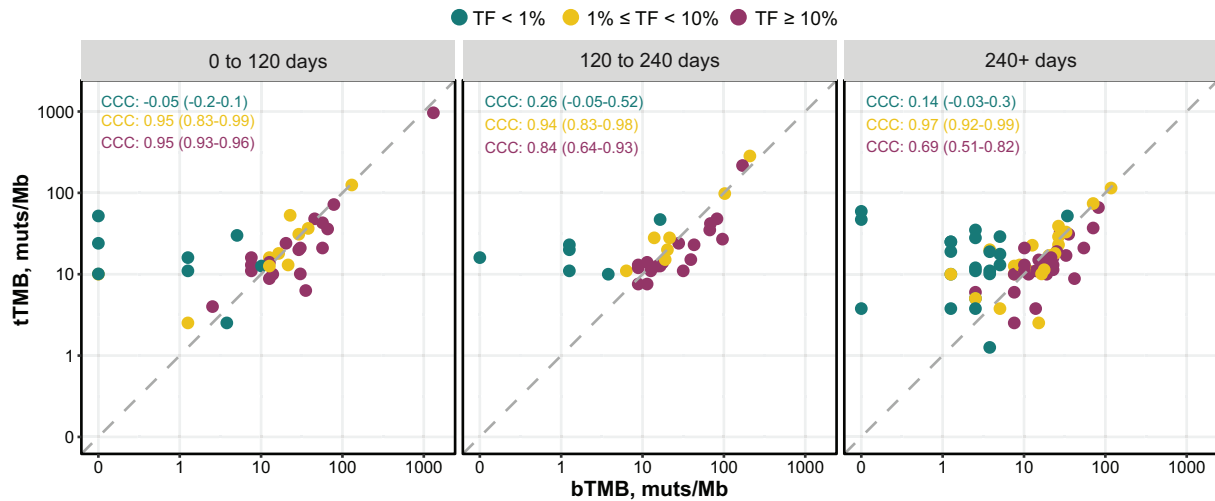**Supplemental Figure 8**

Supplement: Supplementary file 8 — Fig. S8. Correlation between bTMB and tTMB by time between sample collection. (A) Distribution of time between tissue and plasma collection for bTMB and tTMB correlation analysis. (B) bTMB and tTMB remained correlated when TF was >1%, irrespective of time between tissue and plasma collection. ctDNA, circulating tumor DNA; TF, tumor fraction; bTMB, blood tumor mutational burden; mut/Mb, mutations per megabase; tTMB, tissue tumor mutational burden; CCC, Lin's concordance correlation coefficient. [file MOL2-19-3060-s011.pdf]

**A**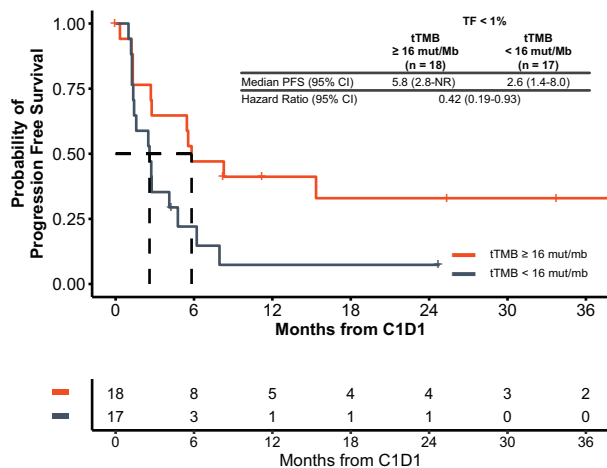**B**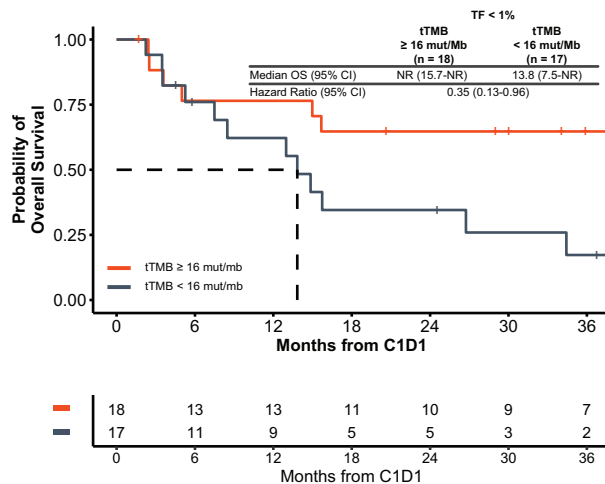**C**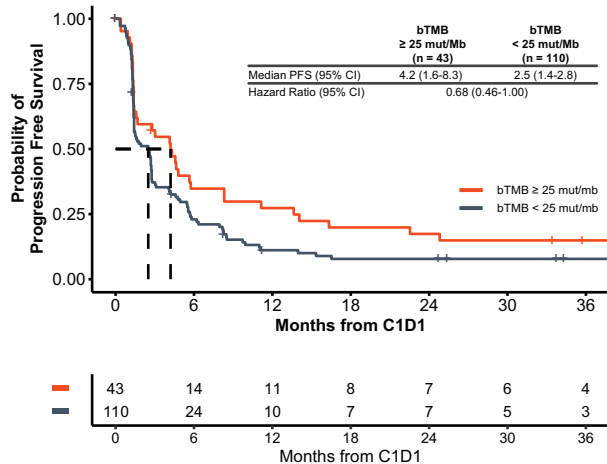**D**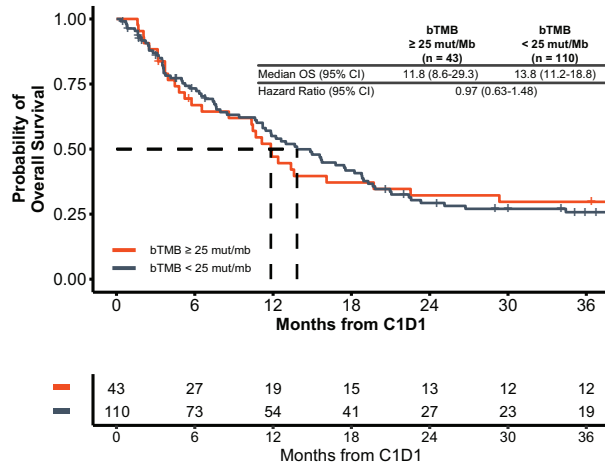

Supplement: Supplementary file 9 — Fig. S9. Outcomes by tTMB status in patients with low ctDNA TF (<1%) and outcomes by bTMB status using a cutoff of 25 mut/Mb. (A, B) In patients with ctDNA TF <1%, where bTMB is not reliable, tTMB ≥16 mut/mb is strongly associated with decreased risk of progression and death. (C, D) In the full treatment cohort, a cutoff of 25 mut/mb was associated with improved PFS, but not OS, in all patients. C1D1, cycle 1 day 1; ctDNA, circulating tumor DNA; TF, tumor fraction; bTMB, blood tumor mutational burden; mut/Mb, mutations per megabase; tTMB, tissue tumor mutational burden; PFS, progression‐free survival; OS, overall survival. [file MOL2-19-3060-s006.pdf]

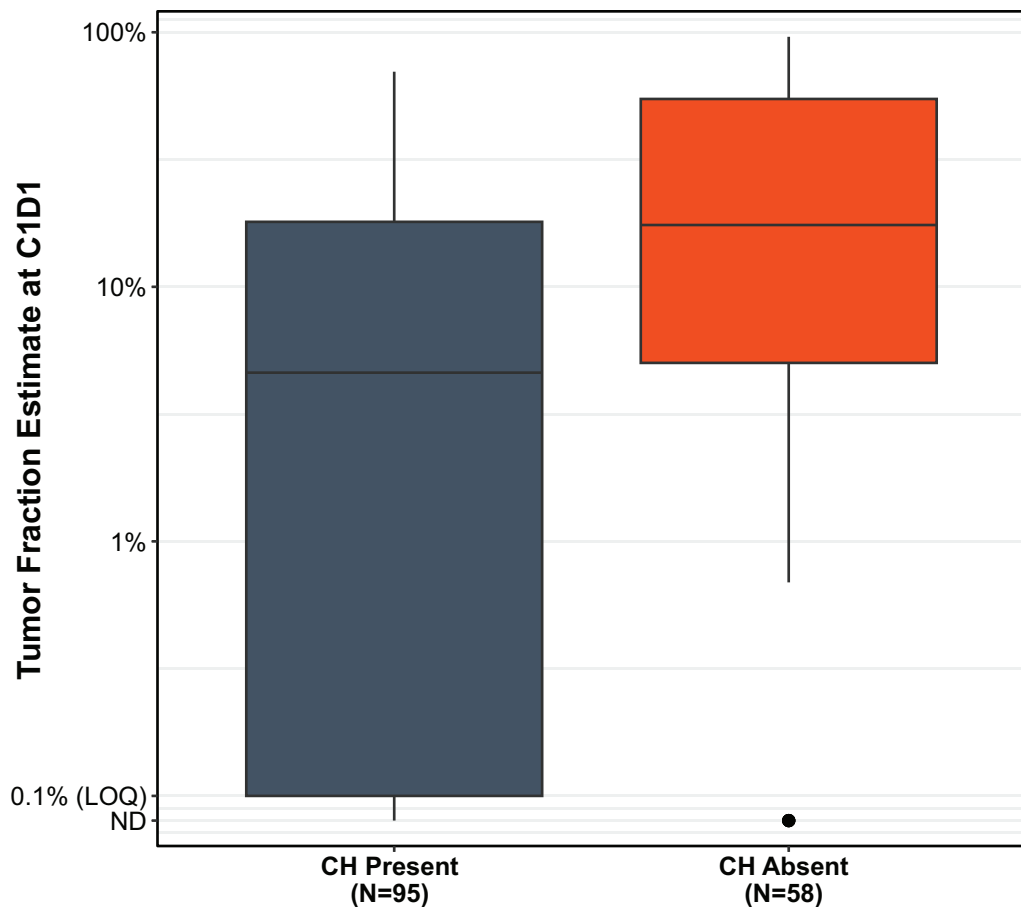

Supplemental Figure 11

Supplement: Supplementary file 11 — Fig. S11. ctDNA TF at C1D1 stratified by the detection of CH alterations. Patients with no predicted CH alterations had higher levels of ctDNA TF. ctDNA, circulating tumor DNA; TF, tumor fraction; CH, clonal hematopoiesis; C1D1, cycle 1 day 1; LOQ, limit of quantification; ND, not detected. [file MOL2-19-3060-s001.pdf]
