# Supplementary material for: Circulating tumor DNA monitoring and blood tumor mutational burden in patients with metastatic solid tumors treated with atezolizumab
Source: Mol Oncol. 2025 May 28;19(11):3060–78. doi: 10.1002/1878-0261.70054 (PMC12591311; doi:10.1002/1878-0261.70054)
Supplement: Supplementary file 10 — Fig. S10. Correlation between maxVAF and ctDNA TF by alteration origin. (A) For all variants, the maximum VAF at C1D1 was moderately associated with ctDNA TF. (B) When predicted germline variants were excluded, a stronger association between maximum VAF at C1D1 and ctDNA TF was observed. C1D1, cycle 1 day 1; ctDNA, circulating tumor DNA; TF, tumor fraction; maxVAF, maximum variant allele frequency; CH, clonal hematopoiesis. [file MOL2-19-3060-s009.pdf]

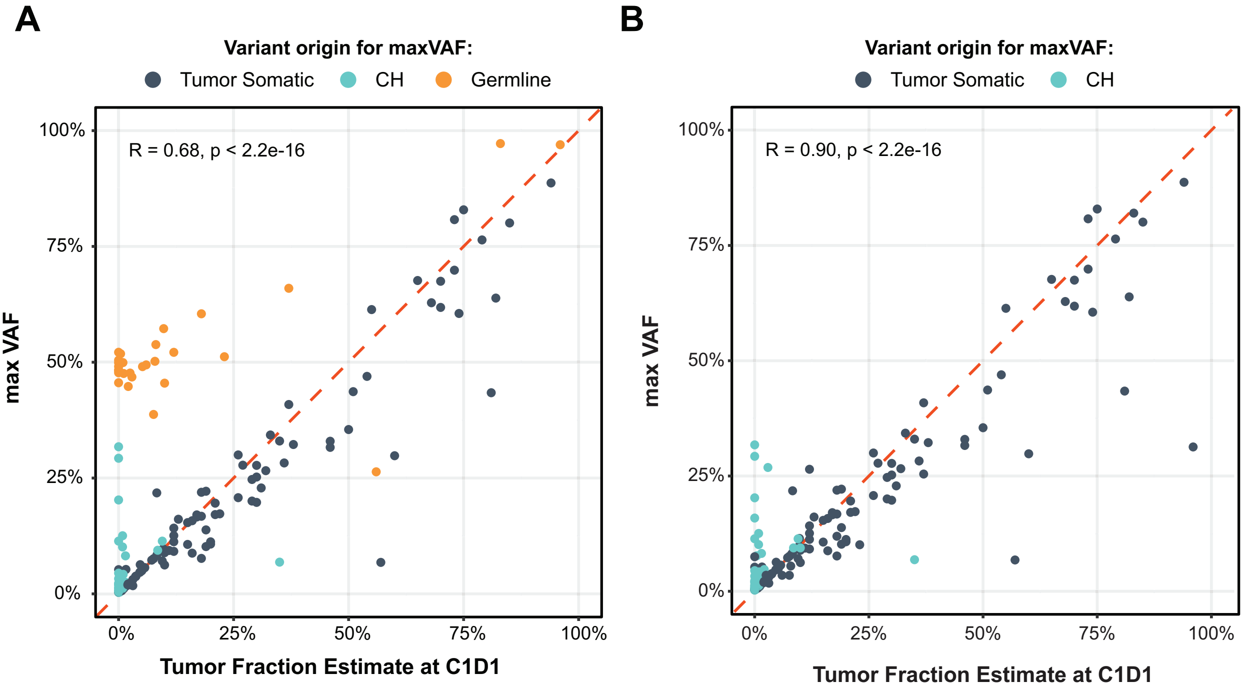

Supplemental Figure 10
