# Supplementary material for: Circulating tumor DNA monitoring and blood tumor mutational burden in patients with metastatic solid tumors treated with atezolizumab
Source: Mol Oncol. 2025 May 28;19(11):3060–78. doi: 10.1002/1878-0261.70054 (PMC12591311; doi:10.1002/1878-0261.70054)
Supplement: Supplementary file 12 — Table S1. Patient characteristics at start of treatment. TF, tumor fraction; C1D1, cycle 1 day 1; IQR, interquartile range; TMB, tumor mutational burden; bTMB, blood TMB, tTMB, tissue TMB; TPS, tumor proportion score. [file MOL2-19-3060-s008.pdf]

Supplemental Table 1: Patient characteristics at start of treatment. TF = tumor fraction, C1D1 = cycle 1 day 1, IQR = interquartile range, TMB = tumor mutational burden, bTMB = blood TMB, tTMB = tissue TMB, TPS = tumor proportion score

	**Overall** N = 153	**Colorectal** N = 34	**Breast** N = 34	**Other Gastrointestinal and Hepatobiliary Tract** N = 27	**Gynecologic** N = 22	**Prostate** N = 7	**Other** N = 29
**ctDNA TF at C1D1 (%), median [IQR]**	8.3 (1.0, 27.0)	6.9 (0.9, 30.0)	9.2 (2.1, 34.5)	7.6 (0.8, 12.0)	15.0 (2.2, 37.8)	18.0 (0.0, 29.0)	7.2 (0.0, 19.0)
**bTMB at C1D1 (mut/mb), median [IQR]**	12.6 (3.8, 27.8)	7.6 (2.5, 19.0)	14.5 (7.6, 19.9)	11.4 (1.9, 20.9)	16.4 (12.6, 28.4)	54.4 (16.4, 82.2)	21.5 (1.3, 35.4)
**tTMB (mut/mb), median [IQR]**	15.0 (11.0, 27.0)	11.4 (8.8, 18.0)	13.0 (11.0, 19.5)	12.6 (10.1, 19.5)	13.2 (10.1, 18.5)	27.0 (18.9, 47.0)	26.5 (18.5, 44.9)
**Age (years), median [IQR]**	67.0 (58.0, 74.0)	63.5 (55.5, 74.8)	61.5 (57.0, 71.0)	66.0 (60.5, 71.0)	67.5 (57.5, 71.8)	80.0 (77.0, 85.0)	70.0 (64.0, 78.0)
**Sex, n (%)**					2		
Female	89 (58.2%)	20 (58.8%)	34 (100.0%)	6 (22.2%)	2 (100.0%)	0 (0.0%)	7 (24.1%)
Male	64 (41.8%)	14 (41.2%)	0 (0.0%)	21 (77.8%)	0 (0.0%)	7 (100.0%)	22 (75.9%)
**Race, n (%)**							
White	116 (75.8%)	28 (82.4%)	22 (64.7%)	20 (74.1%)	15 (68.2%)	7 (100.0%)	24 (82.8%)
Black Or African American	18 (11.8%)	2 (5.9%)	7 (20.6%)	2 (7.4%)	3 (13.6%)	0 (0.0%)	4 (13.8%)
Asian	9 (5.9%)	3 (8.8%)	3 (8.8%)	1 (3.7%)	2 (9.1%)	0 (0.0%)	0 (0.0%)
Native Hawaiian/Other Pacific Islander	1 (0.7%)	0 (0.0%)	0 (0.0%)	0 (0.0%)	1 (4.5%)	0 (0.0%)	0 (0.0%)
American Indian/Alaska Native	3 (2.0%)	0 (0.0%)	1 (2.9%)	1 (3.7%)	0 (0.0%)	0 (0.0%)	1 (3.4%)
Other	6 (3.9%)	1 (2.9%)	1 (2.9%)	3 (11.1%)	1 (4.5%)	0 (0.0%)	0 (0.0%)
**Ethnicity, n (%)**							
Hispanic or Latino	13 (8.5%)	2 (5.9%)	1 (2.9%)	5 (18.5%)	1 (4.5%)	1.0 (14.3%)	3 (10.3%)
Not Hispanic or Latino	133 (86.9%)	31 (91.2%)	31 (91.2%)	20 (74.1%)	21 (95.5%)	6.0 (85.7%)	24 (82.8%)
Not Reported/Unknown	7 (4.6%)	1 (2.9%)	2 (5.9%)	2 (7.4%)	0 (0.0%)	0 (0.0%)	2 (6.9%)
**ECOG, n (%)**							
0	48 (31.4%)	8 (23.5%)	10 (29.4%)	11 (40.7%)	9 (40.9%)	2 (28.6%)	8 (27.6%)
1	99 (64.7%)	26 (76.5%)	22 (64.7%)	16 (59.3%)	12 (54.5%)	4 (57.1%)	19 (65.5%)
2	5 (3.3%)	0 (0.0%)	2 (5.9%)	0 (0.0%)	1 (4.5%)	0 (0.0%)	2 (6.9%)
Not Reported/Unknown	1 (0.7%)	0 (0.0%)	0 (0.0%)	0 (0.0%)	0 (0.0%)	1 (14.3%)	0 (0.0%)
**Prior Systemic Lines of Therapy, n (%)**							
0	10 (6.5%)	0 (0.0%)	0 (0.0%)	1 (3.7%)	0 (0.0%)	0 (0.0%)	9 (31.0%)
1-2	62 (40.5%)	14 (41.2%)	3 (8.8%)	17 (63.0%)	12 (54.5%)	1 (14.3%)	15 (51.7%)
3+	81 (52.9%)	20 (58.8%)	31 (91.2%)	9 (33.3%)	10 (45.5%)	6 (85.7%)	5 (17.2%)
**PD-L1 TPS Status, n (%)**							
<1%	54 (35.3%)	20 (58.8%)	10 (29.4%)	9 (33.3%)	9 (40.9%)	0 (0.0%)	6 (20.7%)
1-49%	11 (7.2%)	1 (2.9%)	1 (2.9%)	1 (3.7%)	2 (9.1%)	1 (14.3%)	5 (17.2%)
50-100%	4 (2.6%)	0 (0.0%)	0 (0.0%)	1 (3.7%)	0 (0.0%)	0 (0.0%)	3 (10.3%)
Not Reported/Unknown	84 (54.9%)	13 (38.2%)	23 (67.6%)	16 (59.3%)	11 (50.0%)	6 (85.7%)	15 (51.7%)
