# Supplementary material for: Circulating tumor DNA monitoring and blood tumor mutational burden in patients with metastatic solid tumors treated with atezolizumab
Source: Mol Oncol. 2025 May 28;19(11):3060–78. doi: 10.1002/1878-0261.70054 (PMC12591311; doi:10.1002/1878-0261.70054)
Supplement: Supplementary file 13 — Table S2. Best confirmed response (cBOR) based of ctDNA tumor fraction at cycle 1 day 1 (C1D1). CR, complete response; PR, partial response; SD, stable disease; PD, progressive disease; ORR, objective response rate (CR + PR); DCR, disease control rate (CR + PR + SD). [file MOL2-19-3060-s015.pdf]

Supplemental Table 2: Best confirmed response based of ctDNA tumor fraction at cycle 1, day 1 (C1D1). CR = complete response. PR = partial response, SD = stable disease, PD = progressive disease, ORR = objective response rate (CR+PR), DCR = disease control rate (CR+PR+SD).

Cohort	ctDNA Tumor Fraction at C1D1	CR	PR	SD	PD	ORR (95% CI)	DCR (95% CI)
All patients	≥1%	4	15	36	56	17% (11%-26%)	50% (40%-59%)
	<1%	3	4	18	13	18% (8%-35%)	66% (49%-80%)
Colorectal	≥1%	1	3	6	14	17% (5%-38%)	42% (23%-63%)
	<1%	2	1	4	2	33% (5%-38%)	78% (40%-96%)
Breast	≥1%	0	2	7	16	8% (1%-44%)	36% (19%-57%)
	<1%	0	0	4	3	0% (0%-44%)	57% (20%-88%)
Other Gastrointestinal and Hepatobiliary	≥1%	1	4	6	7	28% (11%-54%)	61% (36%-82%)
	<1%	0	3	3	3	33% (9%-69%)	67% (31%-91%)
Gynecological	≥1%	2	0	7	12	10% (2%-32%)	43% (23%-66%)
	<1%	0	0	0	1	0% (0%-95%)	0% (0%-95%)
Prostate	≥1%	0	2	1	2	40% (7%-83%)	60% (17%-93%)
	<1%	0	0	2	0	0% (0%-80%)	100% (20% - 100%)
Other	≥1%	0	4	9	5	22% (7%-48%)	72% (46%, 89%)
	<1%	1	0	5	4	10% (1%-46%)	60% (27%-92%)
