# Supplementary material for: Circulating tumor DNA monitoring and blood tumor mutational burden in patients with metastatic solid tumors treated with atezolizumab
Source: Mol Oncol. 2025 May 28;19(11):3060–78. doi: 10.1002/1878-0261.70054 (PMC12591311; doi:10.1002/1878-0261.70054)
Supplement: Supplementary file 14 — Table S3. Patient characteristics for patients who were excluded from outcomes analysis due to missing cycle 3 day 1 (C3D1) liquid biopsy or early progression compared to patients who were included. Response measured by RECIST 1.1. CR, complete response; PR, partial response; SD, stable disease; PD, progressive disease; IQR, interquartile range; TMB, tumor mutational burden; mut/mb, mutations per megabase; bTMB, blood TMB; tTMB, tissue TMB; TPS, tumor proportion score. [file MOL2-19-3060-s003.pdf]

Supplemental Table 3: Patient characteristics for patients who were excluded from outcomes analysis due to missing cycle 3 day 1 (C3D1) liquid biopsy or early progression compared to patients who were included. Response measured by RECIST 1.1. CR = complete response, PR = partial response, SD = stable disease, PD = progressive disease, IQR = interquartile range, TMB = tumor mutational burden, mut/mb = mutations per megabse, bTMB = blood TMB, tTMB = tissue TMB, TPS = tumor proportion score.

	**Included in on-treatment ctDNA detection outcomes analysis**, N = 73	**Excluded from on-treatment ctDNA detection outcomes analysis**, N = 80
**Progressed prior C3D1, n (%)**	0 (0.0%)	58 (72.5%)
**Confirmed Best Overall Response, n (%)**		
CR	7 (9.6%)	0 (0.0%)
PR	15 (20.5%)	4 (5.0%)
SD	48 (65.8%)	6 (7.5%)
PD	3 (4.1%)	66 (82.5%)
Not Reported	0 (0.0%)	4 (5.0%)
**ctDNA TF at C1D1 (%), median [IQR]**	3.1 (0.0, 18.0)	12.0 (3.1, 35.3)
**bTMB at C1D1 (mut/mb) median [IQR]**	10.1 (3.8, 27.8)	14.5 (7.6, 26.9)
**tTMB (mut/mb), median [IQR]**	18.5 (11.0, 33.5)	13.0 (10.1, 20.0)
**Age (years), median [IQR]**	68.0 (59.0, 77.0)	65.0 (56.8, 73.0)
**Sex, n (%)**		
Female	39 (53.4%)	50 (62.5%)
Male	34 (46.6%)	30 (37.5%)
**Race, n (%)**		
White	51 (69.9%)	65 (81.3%)
Black Or African American	12 (16.4%)	6 (7.5%)
Asian	3 (4.1%)	6 (7.5%)
Native Hawaiian/Other Pacific Islander	1 (1.4%)	0 (0.0%)
American Indian/Alaska Native	2 (2.7%)	1 (1.3%)
Other	4 (5.5%)	2 (2.5%)
**Ethnicity, n (%)**		
Hispanic or Latino	6 (8.2%)	7 (8.8%)
Not Hispanic or Latino	61 (83.6%)	72 (90.0%)
Not Reported/Unknown	6 (8.2%)	1 (1.3%)
**ECOG, n (%)**		
0	23 (31.5%)	25 (31.3%)
1	47 (64.4%)	52 (65.0%)
2	2 (2.7%)	3 (3.8%)
Not Reported/Unknown	1 (1.4%)	0 (0.0%)
**Prior Systemic Lines of Therapy, n (%)**		
0	5 (6.8%)	5 (6.3%)
1-2	35 (47.9%)	27 (33.8%)
3+	33 (45.2%)	48 (60.0%)
**PD-L1 TPS Status, n (%)**		
<1%	24 (32.9%)	30 (37.5%)
1-49%	8 (11.0%)	3 (3.8%)
50-100%	3 (4.1%)	1 (1.3%)
Not Reported/Unknown	38 (52.1%)	46 (57.5%)
