# Supplementary material for: Circulating tumor DNA monitoring and blood tumor mutational burden in patients with metastatic solid tumors treated with atezolizumab
Source: Mol Oncol. 2025 May 28;19(11):3060–78. doi: 10.1002/1878-0261.70054 (PMC12591311; doi:10.1002/1878-0261.70054)
Supplement: Supplementary file 15 — Table S4. Best confirmed response (cBOR) based on ctDNA tumor fraction detection at cycle 3 day 1 (C3D1). CR, complete response; PR, partial response; SD, stable disease; PD, progressive disease; ORR, objective response rate (CR + PR); DCR, disease control rate (CR + PR + SD). [file MOL2-19-3060-s012.pdf]

**Supplemental Table 4:** Best confirmed response (cBOR) based on ctDNA tumor fraction detection at cycle 3 day 1 (C3D1). CR = complete response. PR = partial response, SD = stable disease, PD = progressive disease, ORR = objective response rate (CR+PR), DCR = disease control rate (CR+PR+SD).

| Cohort | ctDNA Status | CR | PR | SD | PD | ORR (95%CI) | DCR (95%CI) |
|---|---|---|---|---|---|---|---|
| All patients | Detected at C3D1 | 0 | 10 | 38 | 12 | 17% (9%-29%) | 80% (67%-89%) |
| | Not Detected at C3D1 | 7 | 5 | 11 | 0 | 52% (31%-73%) | 100% (82%-100%) |
| Colorectal | Detected at C3D1 | 0 | 3 | 6 | 3 | 25% (7%-57%) | 75% (43%-93%) |
| | Not Detected at C3D1 | 3 | 0 | 3 | 0 | 50% (19%-81%) | 100% (52%-100%) |
| Breast | Detected at C3D1 | 0 | 1 | 10 | 2 | 8% (0%-38%) | 85% (54%-97%) |
| | Not Detected at C3D1 | 0 | 1 | 0 | 0 | 100% (5%-100%) | 100% (5%-100%) |
| Other Gastrointestinal and Hepatobiliary | Detected at C3D1 | 0 | 1 | 8 | 3 | 8% (0%-40%) | 75% (43%-93%) |
| | Not Detected at C3D1 | 1 | 4 | 1 | 0 | 83% (36%-99%) | 100% (52%-100%) |
| Gynecological | Detected at C3D1 | 0 | 0 | 6 | 2 | 0% (0%-40%) | 75% (36%-96%) |
| | Not Detected at C3D1 | 2 | 0 | 1 | 0 | 67% (13%-98%) | 100% (31%-100%) |
| Prostate | Detected at C3D1 | 0 | 2 | 2 | 0 | 50% (15%-85%) | 100% (40%-100%) |
| | Not Detected at C3D1 | 0 | 0 | 1 | 0 | 0% (0%-95%) | 100% (5%-100%) |
| Other | Detected at C3D1 | 0 | 3 | 6 | 2 | 27% (7%-60%) | 82% (48%-97%) |
| | Not Detected at C3D1 | 1 | 0 | 5 | 0 | 17% (1%-64%) | 100% (52%-100%) |
