# Supplementary material for: Circulating tumor DNA monitoring and blood tumor mutational burden in patients with metastatic solid tumors treated with atezolizumab
Source: Mol Oncol. 2025 May 28;19(11):3060–78. doi: 10.1002/1878-0261.70054 (PMC12591311; doi:10.1002/1878-0261.70054)
Supplement: Supplementary file 16 — Table S5. Patient characteristics for those with persistent negative or unquantifiable ctDNA at cycle 3 day 1 (C3D1). CR, complete response; PR, partial response; SD, stable disease; PD, progressive disease; TMB, tumor mutational burden; mut/mb, mutation per megabase; bTMB, blood TMB; tTMB, tissue TMB; TPS, tumor proportion score; IQR, interquartile. [file MOL2-19-3060-s010.pdf]

Supplemental Table 5: Patient characteristics for those with persistent negative or unquantifiable ctDNA at cycle 3 day 1 (C3D1). CR = complete response. PR = partial response, SD = stable disease, PD = progressive disease, TMB = tumor mutational burden, mut/mb = mutation per megabase, bTMB = blood TMB, tTMB = tissue TMB, TPS = tumor proportion score, IQR = interquartile.

	**Included in ctDNA TF change C1D1 to C3D1 outcomes analysis, N = 51**[1]	**Excluded from ctDNA TF change C1D1 to C3D1 outcomes analysis, N = 22**[1]
**Confirmed Best Overall Response, n (%)**		
CR	5 (9.8%)	2 (9.1%)
PR	12 (23.5%)	3 (13.6%)
SD	31 (60.8%)	17 (77.3%)
PD	3 (5.9%)	0 (0.0%)
Not Reported	0 (0.0%)	0 (0.0%)
**ctDNA TF at C1D1 (%), median [IQR]**	9.8 (2.4, 23.5)	0.0 (0.0, 0.0)
**bTMB at C1D1 (mut/mb), median [IQR]**	16.4 (8.2, 36.7)	2.5 (1.3, 3.8)
**tTMB (mut/mb), median [IQR]**	18.0 (11.0, 35.8)	18.9 (11.0, 29.0)
**Age (years), median [IQR]**	67.0 (58.0, 71.0)	76.5 (64.8, 81.0)
**Sex, n (%)**		
Female	27 (52.9%)	12 (54.5%)
Male	24 (47.1%)	10 (45.5%)
**Race, n (%)**		
White	35 (68.6%)	16 (72.7%)
Black Or African American	8 (15.7%)	4 (18.2%)
Asian	2 (3.9%)	1 (4.5%)
Native Hawaiian/Other Pacific Islander	1 (2.0%)	0 (0.0%)
American Indian/Alaska Native	2 (3.9%)	0 (0.0%)
Other	3 (5.9%)	1 (4.5%)
**Ethnicity, n (%)**		
Hispanic or Latino	4 (7.8%)	2 (9.1%)
Not Hispanic or Latino	43 (84.3%)	18 (81.8%)
Not Reported/Unknown	4 (7.8%)	2 (9.1%)
**ECOG, n (%)**		
0	17 (33.3%)	6 (27.3%)
1	31 (60.8%)	16 (72.7%)
2	2 (3.9%)	0 (0.0%)
Not Reported/Unknown	1 (2.0%)	0 (0.0%)
**Prior Systemic Lines of Therapy, n (%)**		
0	4 (7.8%)	1 (4.5%)
1-2	22 (43.1%)	13 (59.1%)
3+	25 (49.0%)	8 (36.4%)
**PD-L1 TPS Status, n (%)**		
<1%	14 (27.5%)	10 (45.5%)
1-49%	8 (15.7%)	0 (0.0%)
50-100%	2 (3.9%)	1 (4.5%)
Not Reported/Unknown	27 (52.9%)	11 (50.0%)
