# Supplementary material for: Circulating tumor DNA monitoring and blood tumor mutational burden in patients with metastatic solid tumors treated with atezolizumab
Source: Mol Oncol. 2025 May 28;19(11):3060–78. doi: 10.1002/1878-0261.70054 (PMC12591311; doi:10.1002/1878-0261.70054)
Supplement: Supplementary file 17 — Table S6. Best confirmed response based on change in ctDNA tumor fraction detection at cycle 3 day 1 (C3D1). CR, complete response; PR, partial response; SD, stable disease; PD, progressive disease; ORR, objective response rate (CR + PR); DCR, disease control rate (CR + PR + SD). [file MOL2-19-3060-s002.pdf]

Supplemental Table 6: Best confirmed response based on change in ctDNA tumor fraction detection at cycle 3 day 1 (C3D1). CR = complete response. PR = partial response, SD = stable disease, PD = progressive disease, ORR = objective response rate (CR+PR), DCR = disease control rate (CR+PR+SD).

Cohort	ctDNA Change	CR	PR	SD	PD	ORR (95% CI)	DCR (95% CI)
All patients	<50% decrease/increase	0	0	23	3	0% (0%-16%)	88% (69%-97%)
	≥50% decrease	5	12	8	0	68% (46%-84%)	100% (83%-100%)
Colorectal	<50% decrease/increase	0	0	4	2	0% (0%-48%)	67% (24%-94%)
	≥50% decrease	2	2	1	1	67% (24%-94%)	83% (36%-99%)
Breast	<50% decrease/increase	0	0	5	1	0% (0%-48%)	83% (36%-99%)
	≥50% decrease	0	2	1	1	50% (15%-85%)	75% (22%-99%)
Other Gastrointestinal and Hepatobiliary	<50% decrease/increase	0	0	6	2	0% (0%-40%)	75% (36%-96%)
	≥50% decrease	1	3	0	1	80% (30%-99%)	80% (30%-99%)
Gynecological	<50% decrease/increase	0	0	6	1	0% (0%-44%)	86% (42%-99%)
	≥50% decrease	2	0	1	1	50% (15%-85%)	75% (22%-99%)
Prostate	<50% decrease/increase	0	0	0	0	-	-
	≥50% decrease	0	2	1	0	67% (13%-98%)	100% (31%-100%)
Other	<50% decrease/increase	0	0	3	0	0% (0-69%)	100% (31%-100%)
	≥50% decrease	0	3	4	0	43% (12%-80%)	100% (56%-100%)
