# Supplementary material for: Circulating tumor DNA monitoring and blood tumor mutational burden in patients with metastatic solid tumors treated with atezolizumab
Source: Mol Oncol. 2025 May 28;19(11):3060–78. doi: 10.1002/1878-0261.70054 (PMC12591311; doi:10.1002/1878-0261.70054)
Supplement: Supplementary file 18 — Table S7. Best confirmed response stratified by ctDNA tumor fraction (TF) and blood tumor mutational burden (bTMB). CR, complete response; PR, partial response; SD, stable disease; PD, progressive disease; ORR, objective response rate (CR + PR); DCR, disease control rate (CR + OR + SD); CI, confidence interval. [file MOL2-19-3060-s013.pdf]

Supplemental Table 7: Best confirmed response stratified by ctDNA tumor fraction (TF) and blood tumor mutational burden (bTMB). CR = complete response. PR = partial response, SD = stable disease, PD = progressive disease, ORR = objective response rate (CR+PR), DCR = disease control rate (CR+OR+SD), CI = confidence interval.

Cohort	ctDNA TF Status	Blood tumor mutational burden	CR	PR	SD	PD	ORR (95% CI)	DCR (95% CI)
All patients	≥1%	<16 mut/mb	1	1	17	30	4% (1%-15%)	39% 26%-54%)
		≥16 mut/mb	3	14	19	26	27% (17%-40%)	58% (45%-70%)
	<1%	<16 mut/mb	2	4	17	13	17% (7%-33%)	64% (46%-79%)
		≥16 mut/mb	1	0	1	0	50% (9%-91%)	100% (20%-100%)
Colorectal	≥1%	<16 mut/mb	1	1	4	2	25% (4%-64%)	75% (36%-96%)
		≥16 mut/mb	1	0	0	0	100% (5%-100%)	100% (5%-100%)
	<1%	<16 mut/mb	0	0	5	12	0% (0%-23%)	29% (11%-56%)
		≥16 mut/mb	1	3	1	2	57% (20%-88%)	71% (30%-95%)
Breast	≥1%	<16 mut/mb	0	0	4	3	0% (0%-44%)	57% (20%-88%)
		≥16 mut/mb	0	0	0	0	NA	NA
	<1%	<16 mut/mb	0	1	4	6	9% (0%-43%)	45% (18%-75%)
		≥16 mut/mb	0	1	3	10	7% (0%-36%)	29% (10%-58%)
Other Gastrointestinal and Hepatobiliary	≥1%	<16 mut/mb	0	3	3	3	33% (9%-69%)	67% (31%-91%)
		≥16 mut/mb	0	0	0	0	NA	NA
	<1%	<16 mut/mb	0	0	3	6	0% (0%-37%)	33% (9%-69%)
		≥16 mut/mb	1	4	3	1	56% (23%-85%)	89% (51%-99%)
Gynecological	≥1%	<16 mut/mb	0	0	0	1	0% (0%-95%)	0% (0%-95%)
		≥16 mut/mb	0	0	0	0	NA	NA
	<1%	<16 mut/mb	1	0	3	5	11% (1%-49%)	44% (15%-77%)
		≥16 mut/mb	1	0	4	7	8% (0%-40%)	42% (16%-71%)
Prostate	≥1%	<16 mut/mb	0	0	1	0	0% (0%-95%)	0% (5%-100%)
		≥16 mut/mb	0	0	1	0	0% (0%-95%)	0% (5%-100%)
	<1%	<16 mut/mb	0	0	0	0	NA	NA
		≥16 mut/mb	0	2	1	2	40% (7%-83%)	60% (17%-93%)
Other	≥1%	<16 mut/mb	1	0	5	4	10% (1%-46%)	60% (27%-86%)
		≥16 mut/mb	0	0	0	0	NA	NA
	<1%	<16 mut/mb	0	0	2	1	0% (0%-69%)	67% (13%-98%)
		≥16 mut/mb	0	4	7	4	27% (9%-55%)	73% (45%-91%)
